# *Xist* condensates: perspectives for therapeutic intervention

**DOI:** 10.1186/s13059-025-03666-8

**Published:** 2025-07-21

**Authors:** Irene Perotti, Laura Broglia, Gian Gaetano Tartaglia, Andrea Cerase

**Affiliations:** 1 Unit of Cell and Developmental Biology, Department of Biology, Università di Pisa, Strada Statale dell’Abetone Brennero, 4, Pisa, 56123 Italy; 2 RNA Systems Biology Lab, Center for Human Technologies, Istituto Italiano di Tecnologia, Via Enrico Melen, 83, Genoa, 16152 Italy; 3 Blizard Institute, Barts and The London School of Medicine and Dentistry, Queen Mary University of London, London, E1 2AT UK

**Keywords:** Xist, X-chromosome inactivation, Liquid-liquid phase separation, X-reactivation, X-linked disorders, RNA-binding proteins, Condensates, Condensate-modifying therapeutics

## Abstract

X-chromosome inactivation (XCI) is a crucial mechanism of dosage compensation in female mammals ensuring that genes from only one X chromosome are expressed, initiated through expression of the long noncoding RNA *Xist*. Recent evidence underscores the significance of molecular crowding—most likely via liquid–liquid phase separation (LLPS)—in forming *Xist* RNA-driven condensates critical for establishing and sustaining the silenced state. By integrating existing knowledge and emerging ideas, we provide a comprehensive perspective on the molecular underpinnings of XCI and outline how manipulation of LLPS-based mechanisms offers new avenues for novel therapeutic approaches.

**Supplementary Information:**

The online version contains supplementary material available at 10.1186/s13059-025-03666-8.

## Introduction

**Fig. 1:**
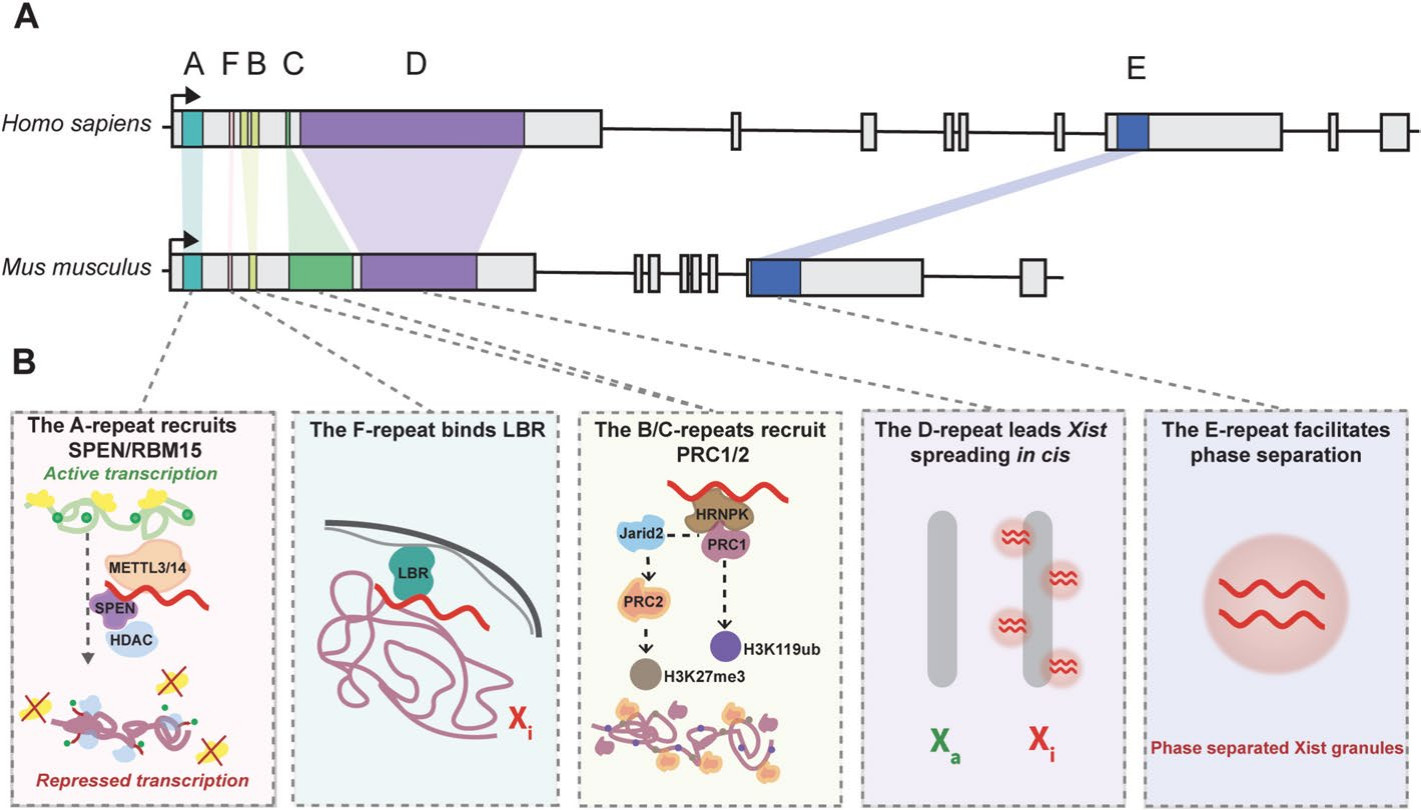
Comparative overview of *Xist* RNA repeat regions in humans and mice. **A** The *Xist* repeats exhibit evolutionary conservation between humans and mice, although most repeat regions vary considerably in copy number. **B** *Xist* (shown in red) comprises five functionally distinct repeat regions whose coordinated activities drive X‑chromosome inactivation (XCI): the A‑repeat at the 5′ end is highly conserved in both sequence and copy number and nucleates XCI initiation by recruiting SPEN to block RNA polymerase II (yellow) and promote removal of histone acetylation marks (green dots); the F‑repeat anchors the inactive X (Xi) to the nuclear lamina via the Lamin B Receptor (LBR), preserving higher‑order chromatin organization and sustained silencing; the B/C‑repeats engage Polycomb repressive complexes PRC1 and PRC2 to deposit H2AK119ub and H3K27me3, reinforcing chromatin compaction and maintenance of gene repression; the D‑repeat directs *Xist* RNA in cis along the Xi chromosome to ensure thorough coating of its target; and the E‑repeat drives phase separation to form *Xist* granules that concentrate silencing factors essential for stable gene silencing

In female mammals, one of the two X chromosomes is epigenetically silenced in all somatic cells through a process known as X-chromosome inactivation (XCI) [1–6]. This process plays a crucial role in maintaining gene dosage parity between the sexes and contributing to regulating X-linked gene expression in female cells [1]. In mouse embryos, XCI occurs during early female development, in the epiblast cells of implanting blastocysts, and is initiated by the expression of the key orchestrator *Xist*. *Xist* is a 15–17-kb long noncoding RNA (lncRNA) transcribed from the X-Inactivation Centre (XIC), a complex *locus* that determines the number and identity of X chromosomes to be inactivated [2, 7] via an interconnected interplay of lncRNAs [8–15], transcription factors [16–19], and chromatin remodelers [20, 21]. During the initiation phase of XCI, *Xist* is expressed and coats the future inactive X chromosome (Xi, Barr body) *in cis*, recruiting various repressive protein complexes through direct and indirect binding mostly to six repetitive elements, within *Xist* RNA Repeats A to F [1] (Fig. 1). The recruitment of these proteins coordinates a series of epigenetic modifications that result in the formation of a compact heterochromatin structure and spatial 3D structural rearrangement of the X chromosome, effectively silencing most genes on the Xi, with the exception of certain escapee genes that remain expressed [22]. Each of these repeats serves a unique function by recruiting specific RNA-binding proteins (RBPs). For instance, the A-repeat plays a crucial role in initiating XCI by recruiting the transcriptional repressor SPEN and RBM15, which facilitate gene silencing through mechanisms like histone deacetylation and N6-methyladenosine (m^6^A) RNA modification, respectively [23]. In contrast, the B/C repeats are indispensable for stabilizing and maintaining the silent state of the X chromosome, as they recruit repressive proteins, through the interaction with HNRNPK, and promote chromatin modifications such as H2AK119ub and H3K27me3, thereby ensuring long-term silencing [24]. The A/E repeats are essential for the accumulation of intrinsically disordered regions (IDRs)-containing proteins on the Xi, establishing functional gradients of silencing factors by means of phase separation [25, 26]. In vitro studies using mouse cells have shown that dimeric *Xist foci* initiate the formation of large protein complexes resembling phase-separated condensates such as paraspeckles in size, shape, and composition, ensuring effective and sustained X-chromosome inactivation [27]. This process is driven by transient homotypic and heterotypic interactions between nucleic acids and IDR-containing proteins, which are key in facilitating liquid–liquid phase separation (LLPS) [27]. Although LLPS was predicted to be the main mechanism for condensate formation and supported by four independent papers [26, 28–30], other mechanisms are possible. Indeed, different types of phase separation can occur—for instance, polymerization-induced microphase separation, gelation, or percolation [31–33].

In both humans and mice, the maintenance of the inactive state of the X chromosome is achieved through a combination of chromatin factors, chromosome conformation, and nuclear compartmentalization [34]. In the context of developing therapies for human X-linked disorders, detailed knowledge of the inactive X chromosome maintenance mechanisms is essential, as they stabilize silencing and present barriers to reactivation strategies. XCI is particularly significant for the expression of X-linked dominant disorders, as X chromosomes can carry mutated alleles. In males, mutations on the single X chromosome are often lethal [35]. In contrast, in females, the same mutation can result in variable phenotypes due to their somatic mosaicism [34]. Some cells express the wild-type (WT) X chromosome, while others express the mutated one [36]. Once XCI occurs, the inactive X chromosome retains an epigenetic memory of silencing, which is typically stable across cell divisions [37, 38]. However, this epigenetic memory is fully reversible at the initial stages of XCI [37, 39]. Reactivation of the inactive X chromosome (XCR) naturally occurs in mice during early development, when many of the repressive chromatin mechanisms on the Xi are either partially or completely erased. In humans, XCR takes place in primordial germ cells (PGCs) and the inner cell mass (ICM), where both X chromosomes are active in female cells, although, whether a true XCR happens after early dampening is still debated [36]. Studies on XCR have provided valuable insights into the mechanisms of repressive chromatin erasure, epigenetic reprogramming, gene silencing stability, and chromatin organization [40]. Understanding X chromosome inactivation can enhance our knowledge of gene regulation and offers potential therapeutic avenues for selectively or semi-selectively reactivating WT alleles on the Xi [36].

The aim of this review is to provide an overview of XCI and its reactivation by describing its fundamental mechanisms and key molecular players, which are crucial for forming and maintaining stable silencing in the Barr body. While we will examine current and emerging strategies for reactivating the Xi, including pharmacological approaches, genetic and epigenetic editing techniques, our review will focus on the modulation of LLPS as a promising new avenue for future therapeutic intervention.

### Key regulatory regions of *Xist* RNA in X chromosome inactivation: mechanisms of gene silencing by *Xist* tandem repeats

Several tandem repeats of the lncRNA *Xist* play a fundamental role in regulating the silencing process. Most mechanistic insights derive from studies in differentiating female mESCs, with growing evidence in human cells indicating a high degree of conservation but also some species-specific nuances. The A-repeat region of *Xist*, located near the 5' end, is essential for recruiting the transcriptional repressor SPEN (SHARP) and RBM15, an auxiliary subunit of the multiprotein complex that catalyzes RNA m^6^A (Fig. 1). Live imaging using mouse neuronal progenitor cells demonstrates that SPEN, a ~ 400 kDa RBP, binds to *Xist* RNA immediately after *Xist* activation, implying that SPEN-driven repression begins in tandem with *Xist* coating [23]. Consistently, in human ESCs, it has also been shown to interact with *XIST* RNA by RIP/CLIP experiments, confirming that this mechanism of binding is conserved between mouse and human systems [41]. SPEN localizes to transcriptionally active promoters and enhancers on the X chromosome and initiates silencing by associating the histone deacetylase complexes (NURD, NCOR/SMRT), which also includes HDAC3, through its SPOC domain [23, 42]. The action of these repressive complexes strongly reduces the accessibility of RNA polymerase II to the chromatin, thereby preventing gene transcription [42–45] (Fig. 1). Counterintuitively, at the early stages of XCI, before gene silencing occurs, nucleosomes at promoters become less compact, a process that relies on the BAF complex [21, 23]. In mouse ESCs, depletion of key BAF components such as SMARCC1 or SMARCA4 prevents *Xist* spreading, polycomb-associated repressive marks are not established, and gene silencing is impaired [21, 23]. Once gene silencing is established, nucleosomes condense again, and SPEN dissociates from chromatin and it is no longer required for silencing maintenance, indicating that its role is primarily in the establishment of XCI [21, 23], including proper *Xist* RNA upregulation at the onset of XCI [46].

RBM15 through direct interaction with *Xist* RNA [47] recruits the m^6^A RNA methylation machinery, particularly the METTL3/14 complex, which is responsible for modifying *Xist* RNA [48] (Fig. 1). These events are critical for *Xist* functionality in silencing, reinforcing the importance of the A-repeat for successful XCI initiation. Wutz et al. further highlights this by demonstrating in mouse that a 0.9 kb deletion at the 5' end of the lncRNA, encompassing the A-repeat region, completely abolishes its silencing activity [39] (Fig. 1).

The Repeats B/C are essential for preserving the silent state of the X chromosome through the recruitment of repressive proteins such as HNRNPK (Heterogeneous Nuclear RiboNucleoProtein K) (Fig. 1). In mouse, HNRNPK directly binds this cytosine-rich region mediating the recruitment of the non-canonical Polycomb repressive complex 1 (PRC1) [24] adorning the Xi chromosome with a single ubiquitin molecule to histone H2A at Lys119 (H2AK119ub1) [39, 48–50]. In human fibroblasts, HNRNPK associates with the equivalent B/C-proximal region of *XIST* [51]. The accumulation of H2AK119ub mirrors the pattern of *Xist* spreading, starting near the *Xist locus* and extending to gene-dense areas that are in topological contact with *Xist* entry sites and eventually reaching nearby intergenic regions [52–57]. This chromatin modification is fundamental for the recruitment of Jarid2, the cofactor of the Polycomb repressive complex 2 (PRC2), which catalyzes mono-, di-, and tri-methylation of histone H3 at Lys27 (H3K27me1, H3K27me2, and H3K27me3, respectively) [58, 59]. PRC1 and PRC2 localize to the same genomic regions in most instances, creating Polycomb chromatin domains [60, 61] (Fig. 1). Bowness et al. recently compared the roles of SPEN and PRC1, which are both involved in the initial XCI stages and recruited by different *Xist* repeats. These proteins work cooperatively in gene silencing and disruption of either pathway led altered XCI [62]. SMCHD1, a protein that accumulates on the Xi about 3–4 days after *Xist* induction in mouse, relies on H2AK119ub but not H3K27me3 for its recruitment [62–65] (Fig. 1). Although its role seems crucial during XCI establishment, it is not essential for maintaining gene silencing once established, as its removal does not reactivate many genes. Indeed, SMCHD1 primarily silences genes repressed later in XCI and requires differentiation for its function [62, 66, 67]. Interestingly, while the A-repeat plays a crucial role in the initiation of XCI, the B/C repeats seem to be fundamental for the stabilization and maintenance of the heterochromatinization of the Barr body, and the lack of these regions leads to changes in the chromatin organization of the Xi in the nucleus heading defects in the silencing of most of its genes [68, 69].

The E-repeat region of *Xist* is critical for directing *Xist* to the future Xi [28]. In mouse, the absence of this region leads to gene reactivation and decrease of PcG marks during the maintenance phase of X chromosome inactivation [28]. Several proteins, including the nuclear matrix protein CIZ1 (CDKN1A-Interacting Zinc Finger Protein 1), bind to the E-repeat, playing a key role in confining *Xist* RNA molecules to the X chromosome [28, 70, 71]. Additionally, proteins such as PTBP1, MATR3, CELF1, and TDP-43 organize into specific functional clusters that appear to contribute to efficient *Xist*-mediated gene silencing forming phase-separated nuclear compartments [27, 28] (see “Phase-separated Xist condensates” paragraph) (Fig. 1).

The F-repeat region contains multiple binding sites for YY1 (Yin and Yang 1), a transcription factor crucial for regulating *Xist* expression [20, 72]. *Xist* interaction with LBR (Lamin B receptor) is also important [52–54, 73, 74] (Fig. 1). It has been demonstrated, using an inducible *Xist* in male ESCs, that deleting this region significantly disrupts transcriptional silencing by preventing the efficient tethering of the Xi to the LBR at the periphery of the nucleus [72, 75–77]. Although an interaction between *Xist* RNA and LBR was proposed to be sufficient for its spreading during X-chromosome inactivation [77], other studies show only minor silencing defects upon its depletion, suggesting that it may not strictly required for XCI [78, 79]. Thus, while LBR may help to establish and stabilize gene repression, its precise role in XCI remains uncertain, due LBR multiple cellular functions.

The D‑repeat of *Xist*, comprising multiple copies of a 290‑nucleotide motif, serves as the primary binding site for scaffold attachment factor A (SAF‑A/Hnrnpu), which is essential for broad localization of *Xist* RNA to the Xi [80, 81]. However, Repeat D may not act alone in ensuring *Xist* localization [82]. Evidence indicates that multiple regions of *Xist* likely contribute synergistically or redundantly to its targeting, leaving open the question of whether Repeat D is sufficient or additional elements are required [83]. Deletion studies using CRISPR/Cas9 have highlighted the importance of Repeat D in regulating *Xist* expression and silencing X-linked genes. The absence of Repeat D results in reduced *Xist* levels and upregulation of X-linked genes, underscoring its crucial role in *Xist* functionality and the maintenance of X chromosome inactivation [84] (Fig. 1).

To summarize, XCI is a complex process that involves multiple regions of the Xist RNA, each responsible for different aspects of gene silencing and chromatin organization. The A-repeat initiates gene silencing, while the B/C repeats, although also involved in the initiation, mostly maintain the silent chromatin state. The E-repeat ensures proper recruitment of repressive proteins to *Xist* RNA, and the F-repeat anchors the Xi to the nuclear periphery through interactions with LBR, maintaining the long-term stability of the inactivation process.

### 3D chromosome architecture and gene silencing during X chromosome inactivation

**Fig. 2:**
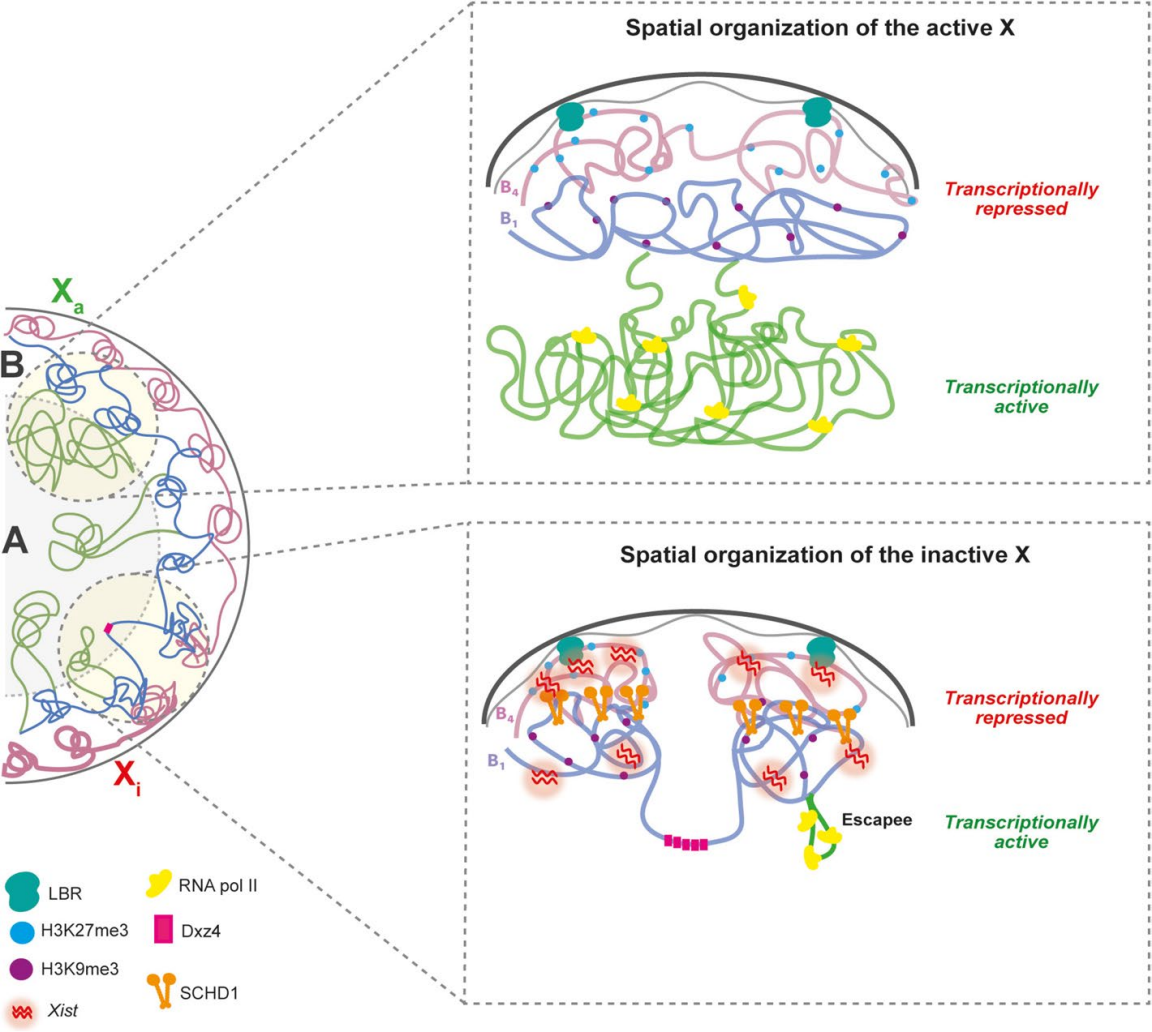
Spatial organization of the active and inactive X chromosome in the nucleus. Chromosomes are organized into distinct territories within the nucleus and are further divided into compartments (right), A compartment (green), which contains highly transcribed regions and B compartment which comprises heterochromatin and is divided into B1 (in blue, characterized by an abundance of Polycomb mark H3K27me3) and B4 subcompartment (in pink, tethered to the nuclear lamina). Top. The active X chromosome exhibits a chromatin arrangement dominated by A compartments (euchromatin, green), which allow RNA polymerase II to transcribe genes efficiently. Additionally, B compartments (heterochromatin, pink and blue) are present, although less prominent. Bottom. The spatial configuration of the inactive X chromosome (Xi) differs significantly. During differentiation, certain domains from the active X shift from the A compartment into the B compartment. The newly formed Xi predominantly adopts a repressed, B-like structure. In the early stages of X chromosome inactivation (XCI), *Xist* RNA selectively coats active regions, recruiting them into a B1-like subcompartment via Polycomb-associated chromatin modifications. Subsequently, SMCHD1 is recruited to the Xi, facilitating the partial merging of B subcompartments. This allows *Xist* RNA to further spread into the B4-like subcompartment, resulting in nearly complete silencing of the X chromosome. Escape regions, however, remain in the A-like compartment, preserving euchromatin and forming large chromatin loops

The inactive X chromosome is only approximately 1.2 times more compressed than the active X chromosome in both mice and humans [85, 86]. Originally, the Barr body was identified near the peri-nucleolar region of the nucleus [87–89]. In addition, more recent studies, both in mouse and human, have shown that the Xi is typically located at the periphery of the nucleus [90–93]. Although peripheral localization seems not to be strictly required for XCI initiation, it might play a role in maintenance of gene silencing [79, 94, 95]. It exhibits a distinct heterochromatic landscape and undergoes significant 3D reorganization during XCI, highlighting the close relationship between 3D chromosome structure and gene expression regulation [96]. These structural differences between the Xa and Xi territories have been long observed. Hi-C analysis shows that the Xi is structured into two large megadomains, separated by the Dxz4 region, which is abundant in CTCF-binding sites unique to the Xi due to its lower DNA methylation (Fig. 2).

This region, along with other elements like *Xist* and *Firre*, forms long-range chromatin interactions known as superloops [75, 96–99]. Unlike the Xa, which is organized into over 100 topologically associated domains (TADs) throughout its length, similarly to the escapee genes [97], the Xi lacks these large-scale structures and instead features frequent intrachromosomal contacts [100]. The boundary between the two megadomains on the Xi, located near the microsatellite repeat DXZ4 in humans or Dxz4 in mice, is conserved across species. While mega-domains rely on Cohesin for their structural organization, super loops function independently of it [75, 96–99]. Though these mega-domains are conserved in mice and humans, they are not crucial for XCI or gene escapee [63, 98, 101]. Deletion or inversion of Dxz4 disrupts the bipartite structure but does not significantly affect gene silencing [98]. Despite its highly condensed, heterochromatic state, the Xi retains a compartment-like organization similar to the A/B compartments (A, transcriptionally active and B, transcriptionally repressed) of the Xa, albeit in a larger and less sharply defined form, both mice and humans. The Xi B compartment is divided into B1- and B4-like subcompartments marked by distinct histone modifications—B1 with H3K27me3 and *Xist* coating, and B4 with H3K9me3 and nuclear lamina association [102, 103] (Fig. 2). The SMCHD1 protein merges these subcompartments, assisting *Xist* in spreading and stabilizing the silenced state of genes [63]. During XCI, *Xist* RNA first coats the X chromosome, triggering gene silencing and leading to the formation of distinct B subcompartments, characterized by the loss of RNA polymerase activity [63, 67, 86]. At this early stage, YY1 binds the nucleation site in exon 1 of the *Xist* gene—anchoring the RNA to the DNA of the inactive X chromosome—and its displacement from Xi has been shown to delay the onset of silencing [104]. This binding is crucial for the lncRNA to remain attached and spread along the chromosome *in cis* [75]. As gene silencing progresses, SMCHD1 spreads across these compartments, ensuring the stable silencing of genes (Fig. 2). Without SMCHD1, some genes fail get silenced properly, and the silencing defect results in female-specific embryonic lethality of *SmcHD1* mutant mice [63, 67, 69]. Additionally, the Xi undergoes reorganization at the sub-megabase level, with attenuated TADs, which are maintained only in expressed regions like *Xist* and escape genes. SMCHD1 plays a crucial role in this process by reducing CTCF and cohesin occupancy, maintaining the compact chromatin structure of the Xi. The Xi forms a heterochromatic Barr body, largely composed of B subcompartments and reliant on SMCHD1 for full gene silencing and chromatin condensation [63, 67, 97].

### Mechanisms of XCI stability

The maintenance of XCI is a multifaceted process involving a range of factors working in concert to ensure the stable and heritable silencing of one X chromosome [39]. Understanding the interplay of these factors (e.g., *Xist* RNA, repressing factors and RBPs) is essential for unraveling the intricacies of XCI maintenance and stability. Among these components, histone modifications are particularly central to the process.

Polycomb repressive complexes, PRC1 and PRC2 are important for XCI maintenance. PRC1 contributes to chromatin compaction and transcriptional repression, while PRC2 contributes by modifying histones to further stabilize the silent state. These complexes ensure that the Xi remains repressed by modifying chromatin and preventing gene expression [39]. In particular, H2AK119ub and H3K27me3 play essential roles in altering the chromatin structure, rendering it less accessible for transcription and reinforcing the silent state of the Xi [105–107].

Another significant factor is the histone variant macroH2A, which replaces the standard histone H2A during XCI [108–110]. While its role in maintaining XCI is somewhat debated, mouse studies indicate that its deletion in late differentiation does not severely disrupt XCI, as female mice lacking this variant are viable and fertile [108–110]. MacroH2A can also be monoubiquitinated, but the relevance of this modification in XCI remains unclear [105]. Nonetheless, the presence of this variant suggests it may play a role in both establishing and stabilizing XCI [105, 108]. DNA methylation is another crucial layer in XCI maintenance. During the inactivation process, CpG islands associated with promoters on the Xi become hypermethylated [64, 111–113]. This hypermethylation correlates with gene silencing and is regulated by the DNA methyltransferase enzyme DNMT3B. In contrast, gene bodies and gene-poor intergenic regions on the Xi are hypomethylated, with DNA methylation at CpG islands being essential for maintaining the inactive state [21, 114–118]. *Xist* RNA is fundamental in initiating XCI, but its role diminishes over time. *Xist* is primarily involved in the establishment of XCI and it also contributes to the transition from *Xist*-dependent to *Xist*-independent silencing [39]. However, conditional *Xist*-knockout studies in mice revealed that the effect of *Xist* deletion on gene reactivation is tissue-dependent, with most tissues showing only limited re-expression from the Xi, whereas blood and gut exhibited severe oncogenic outcomes upon *Xist* loss [73, 119–121].It is unclear whether SPEN remains in the Xi compartment after XCI initiation; however, its presence may explain the maintenance of silencing in the absence of *Xist* before DNA methylation is established [122].

### Phase-separated *Xist* condensates

**Fig. 3:**
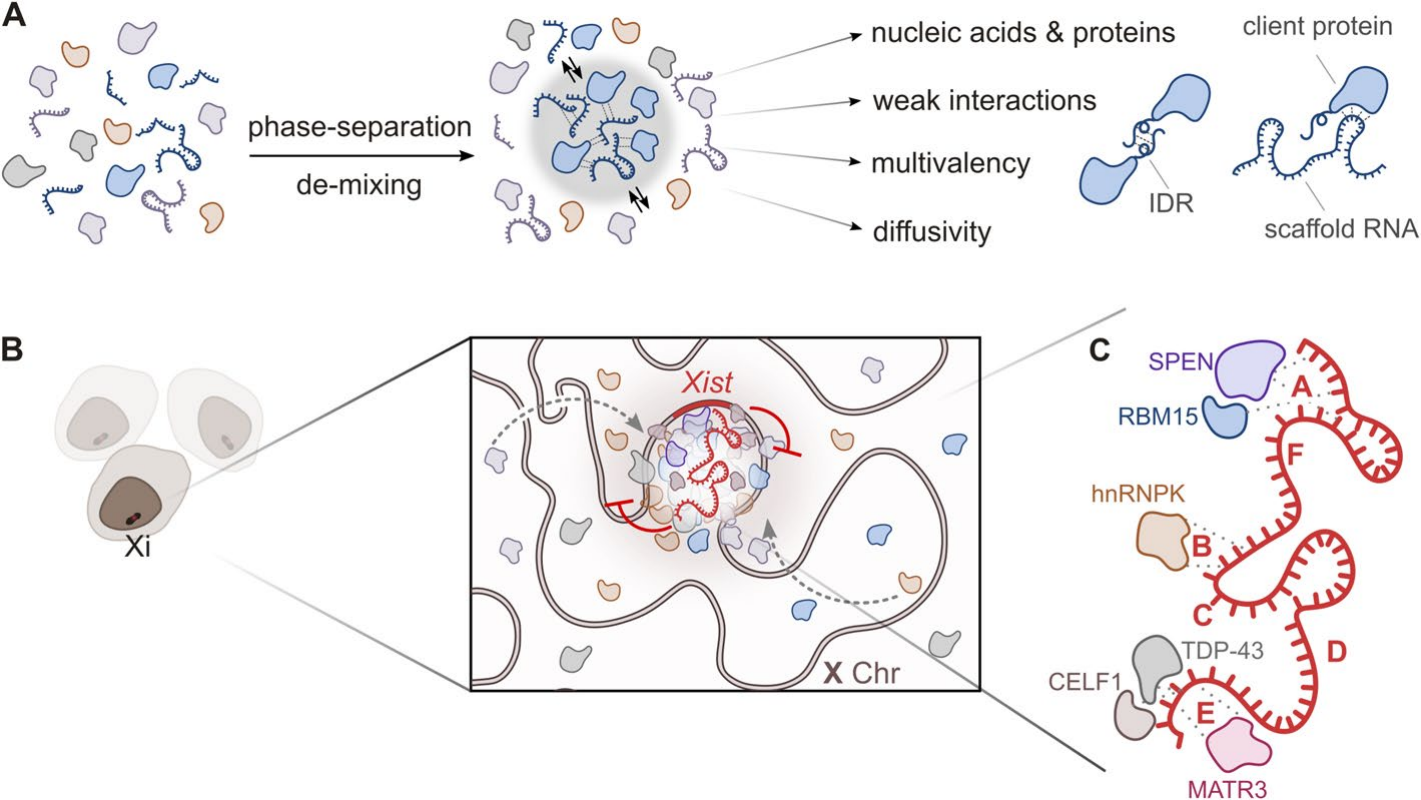
*Xist*-mediated phase separation and protein recruitment on the inactive X chromosome (Xi). **A** Mechanism of phase separation driven by weak, multivalent interactions between nucleic acids and proteins. These interactions facilitate the formation of membrane-less condensates through phase separation. Condensate-promoting features are illustrated. Scaffold RNAs, such as *Xist*, initiate condensate assembly by recruiting proteins containing intrinsically disordered regions (IDRs), resulting in dynamic, reversible molecular environments. **B** Illustration of *Xist* RNA localization to the Xi in a population of cells, emphasizing its role in molecular crowding and the formation of *Xist* condensates. **C** Detailed view of *Xist* RNA with its characteristic repeats (A–F) interacting with various proteins known to harbor iIDRs. Key protein interactors include SPEN, RBM15, HNRNPK, TDP-43, CELF1, and MATR3. Dashed lines represent specific RNA–protein interactions mediated by the *Xist* transcript

LLPS is a process that occurs when the concentration of specific molecular components increases to the point of creating a supersaturated solution, which separates into distinct phases [32, 123, 124] (Fig. 3A). LLPS is widely used in cells to form condensates, i.e., membrane-less organelles with specialized functions. This compartmentalization creates a microenvironment that enhances the efficiency of biochemical reactions occurring within the condensates and while their fluid nature allows for the exchange of molecules with the surrounding environment [125, 126] (Fig. 3A). To date, several membrane-less organelles have been characterized, including stress granules (SGs) that store translationally arrested mRNA and RBPs in response to stress signals; P-bodies, specialized in RNA metabolism and storage; and paraspeckles, nuclear structures involved in gene expression regulation and transcription machinery assembly [126–128]. The formation of phase-separated condensates relies on numerous weak, noncovalent homotypic and heterotypic interactions between nucleic acids and proteins, resulting in the exclusion of solvent molecules (Fig. 3A). The interactions governing partitioning into condensates typically include hydrophobic, electrostatic, π–π stacking, and π–cation forces [32]. Proteins with IDRs, modular protein repeats, and nucleic acids characterized by multivalent binding domains are commonly found in these assemblies [32]. Studies of membrane-less organelles indicate that intrinsic disorder underpins a wide array of molecular interaction networks [129]. Molecules within these condensates can often be categorized into “scaffolds,” which nucleate and maintain the phase-separated state, and “clients,” which are recruited or excluded based on their specific interactions with scaffold components [126, 130, 131].

A key question in the XCI field is how a relatively small number of *Xist foci* [132, 133] can regulate a vast number of X-linked genes across the entire chromosome through the establishment of large macromolecular complexes [32] (Fig. 3B and C). *Xist* recruits a high number of proteins that was predicted to undergo LLPS [27, 134] (Fig. 3B and C). Each repeat element in *Xist* comprises small tandem repeats that confer specific functional properties by attracting distinct RBPs [135]. The defined secondary structures of Repeats A, B, and E, along with the partially structured Repeat D and the predominantly single-stranded 3′ region of *Xist* [27, 136], are involved in the selective recruitment of RBPs. These structural features create binding sites and conformations that enhance the recruiting of RBPs, facilitating the assembly of large multiprotein complexes through extensive protein–protein and protein-RNA interactions [32]. Furthermore, many proteins that interact with *Xist* not only contain disordered regions, which contribute to the dynamic nature of these interactions, but have been reported to undergo phase separation in SGs and paraspeckles (e.g., TDP-43, HNRNPK, CELF1, HnRNPR and RBM14) [27] (Fig. 3B and C). *Xist* repeat regions attract RBPs (Fig. 3B), resembling the NEAT1 lncRNA, which sequesters proteins and drives paraspeckle formation through LLPS [137].

Based on these observations, it was proposed that the recruitment of proteins by *Xist* drives the formation of condensates with increased molecular crowding, facilitating efficient and stable X-chromosome inactivation [27, 134] (Fig. 3B). This hypothesis was successively validated by experimental studies [26, 29, 30]. Standard fluorescence microscopy revealed that *Xist* and its interacting proteins form large complexes, observed as foci distributed across the X chromosome [52, 138]. Remarkably, only 50 to 150 foci were detected on the Xi, raising questions about how a relatively small number of complexes could control the expression of numerous X-linked genes [70, 133, 139]. With the development of quantitative super-resolution microscopy methodologies, researchers were able to demonstrate that *Xist foci* drive the formation of membrane-less compartments that extend across the X chromosome [26, 133]. These nuclear compartments, also called supramolecular complexes (SMACs), are highly dynamic structures that depend on transient, multivalent interactions between *Xist*-binding proteins. Three-dimensional structured illumination microscopy measurements of distances among various *Xist* interactors—SPEN, PCGF5, CELF1, and CIZ1—revealed significantly shorter distances when these proteins are associated with *Xist* compared to the rest of the nucleus [26]. The recruitment of proteins by *Xist* results in a local increase in protein concentration relative to the surrounding nuclear environment. Protein accumulation within the SMAC during the Xi transition varies depending on their specific roles. For instance, SPEN shows an increase in concentration, while CELF1 decreases, and others, such as PTBP1 and HNRNPK, remain stable. This observation suggests that an increase in concentration of certain proteins (i.e., SPEN) is critical for function (i.e., gene silencing), whereas for other proteins, their recruitment at basal levels is sufficient to fulfil their roles. The SPEN IDR domain is essential not only for SPEN recruitment to SMACs but also for the formation of dynamic condensates. Another study also demonstrated that SPEN can form concentration-driven clusters in the nucleus through homotypic multivalent interactions mediated by its IDR domain [29]. SPEN binding to *Xist* via its RNA-binding protein domains appears necessary for its accumulation through the IDR, suggesting that RNA–protein interactions are also crucial in driving this molecular crowding [26]. Experiments utilizing single-cell RNA sequencing and RNA FISH demonstrated that, in the absence of the SPEN IDR domain, silencing of X-linked genes is lost. This finding indicates that this domain is crucial for the regulation of gene expression mediated by SPEN across the entire chromosome [26, 29]. Similarly, the direct binding of SPEN to *Xist* A-repeat through its RRM domains is essential for the proper functioning of SPEN in gene silencing [29]. On the one hand, SPEN spatial amplification explains how *Xist* achieves gene silencing across the entire X chromosome. On the other hand, the low levels of *Xist* expression ensure that this silencing remains specific to the X chromosome and does not extend to autosomes. It has been proposed that a feedback regulatory loop, governed by *Xist-*SPEN, maintains *Xist* levels low, thereby preserving the specificity of its function [29]. However, other mechanisms beyond the *Xist*-SPEN regulatory feedback loop were described to maintain the Xist expression at the appropriate levels [20, 46, 140].

It should be stressed that molecular crowding on Xi likely involves more than just the A-repeat and SPEN. For example, the B-repeat region appears to play a significant role in the accumulation of SMACs by recruiting key factors like PRC1 and SMCHD1. Yet, the E-repeat recruits several proteins that are capable of undergoing phase separation (e.g., PTBP1, CELF1 and TDP-43). The proteins assemble on the E-repeat and, through homotypic and heterotypic protein–protein and RNA–protein interactions, form large higher-order assemblies. E-repeat are loosely characterized by structured regions interspaced by linear RNA portions [141]. Interestingly, linear RNAs within condensates are disordered, allowing them to form multiple interactions with RNAs and to recruit disordered proteins [129]. *Xist* harbors regions that are expected to be linear [27, 142], and they might contribute to build the network of interactions required for the condensate formation.

Moreover, it was recently revealed that *Xist*’s B-repeat and the RGG domain of HNRNPK cooperatively drive LLPS, forming condensates that engage with *Xist* RNA and may facilitate its internalization and spreading along the Xi territory [30]. Repeat B enables HNRNPK to phase separate at lower physiological concentrations and induces a further phase transition that makes the droplets more dynamic, by changing its material properties. These changes enhance condensates ability to entrap silencing factors such as SPEN, YY1, SMCHD1, and RING1B, all of which contain IDRs. In addition, these alterations promote the entrapment of silencing factors and facilitate the spreading of *Xist* within HNRNPK-organized chromosomes, enabling efficient cis-limited inactivation across the X chromosome [30]. Importantly, the study from the Lee lab, demonstrated that a mutant version of HNRNPK defective in LLPS but still able to bind RNA, fails to support proper *Xist* spreading, providing strong evidence for the functional importance of phase separation in vivo. These findings indicate that LLPS plays a crucial role not only in the initiation and maintenance of silencing but also in facilitating its effective spreading across chromosome [143].

In the nuclear environment—where RNA concentration is higher than in the cytoplasm—various condensates, such as paraspeckles, speckles, Cajal bodies, and nucleoli, form through RNA–RBP interactions [144]. *Xist* granules may represent an additional nuclear compartment that helps compartmentalize specific biochemical processes, thereby enhancing their efficiency.

The formation of *Xist*-containing condensates and their role in gene silencing have been primarily studied in mouse systems, with limited functional evidence available from human cells (Table 1). However, we envision that similar molecular organizations exist in the human context. For example, the A-repeat, which contributes to the formation of SMACs, is highly conserved between human and mouse in both copy number and sequence consensus [145], suggesting a conserved structural role in XCI. Furthermore, recent work has shown that SPEN, a key effector in mouse XCI, also associates with *XIST* in naïve human embryonic stem cells and is required for dampening gene expression [41]. Interestingly, proteins known to bind the E-repeat of *Xist* in mouse ESCs—namely PTBP1 and MATR3—have also been implicated in the maintenance of XCI in human cells. In adult human B cells, these factors were found to be important for the repression of the X-linked immune gene *TLR7*, suggesting a possible role in repression maintenance in adult human cells [146]. Given the described roles for E-repeat in the maintenance of gene silencing and *Xis*t localization [28], these observations raise the possibility that similar mechanisms may operate in human cells, with potential species-specific differences yet to uncover. Notably, MATR3 harbors an IDR, further supporting the idea that E-repeat–binding proteins may contribute to XCI maintenance through phase separation–related mechanisms.

**Table 1:** Summary of key studies on *Xist*-mediated condensates and their associated cellular models. This table summarizes key studies investigating the formation and function of *Xist*-mediated condensates, detailing the cell types used, species of origin, main findings, and whether human cell systems were included. Abbreviations: ESCs, embryonic stem cells; MEFs, mouse embryonic fibroblasts; 3D-SIM, three-dimensional structured illumination microscopy; U2OS, human osteosarcoma cell line; HEK293T, human embryonic kidney cell line; SMACs, supramolecular complexes; LLPS, liquid–liquid phase separation; Xi, inactive X chromosome

Study	Cell type(s)	Species	Main finding	Human cell system contribution
Pandya-Jones et al*.,* *Nature* 2020 [28]	- ESCs, - Female MEFs, - Male tet-inducible *Xist*^*ΔTsix*^ V6.5 ES cells	Mouse	E-repeat–binding proteins form a condensate on the Xi that is essential for *Xist* anchoring, gene silencing, and its maintenance (mouse)	Human PTBP1 and CELF1 used in droplet assay
Markaki et al*.,* *Cell* 2021 [26]	- Female ESCs, - Epiblast-like cells - Fibroblasts	Mouse (ESCs) Human (Fibroblasts)	*Xist* seeds local protein gradients to form SMACs, enabling chromosome-wide silencing through spatial concentration of repressive complexes (mouse)	Human fibroblasts used in 3D-SIM to study the variability of *Xist foci* number
Jachowicz et al*.,* *Nat Struct Mol Biol* 2022 [29]	- Female ESCs, - HEK293T	Mouse (ESCs) Human (HEK293T)	*Xist* amplifies SHARP recruitment via concentration-dependent assemblies to achieve chromosome-wide silencing while maintaining specificity (mouse)	SHARP biochemical and biophysical properties studied in human HEK293T cells
Ding et al*., Cell* 2025 [30]	- ESCs, - MEFs, - U2OS cells	Mouse (ESCs MEFs) Human (U2OS)	B-repeat and HNRNPK co-drive LLPS to encapsulate the X chromosome, enabling local concentration and cis-limited spreading of silencing factors (mouse)	HNRNPK phase separation studied in human U2OS cells

### The Xi and X-linked genetic disorders

XCI is not always perfectly random. Skewing has been reported in several X-linked disorders [147, 148]. The direction and extent of XCI skewing (i.e., imbalance in maternal and paternal X silencing) significantly shape the severity of diseases influenced by X chromosome inactivation [147, 148]. In female carriers of X-linked mutations, the X chromosome bearing the mutation can be selectively inactivated, thereby preserving the function of the normal X chromosome and ensuring proper autosomal dosage [149, 150]. However, this skewing is not always consistent; in cases where it is limited or absent, an additional pathogenic variant on the second X chromosome can result in the expression of one of the detrimental mutations, leading to variable disease severity [151].

XCI also plays a critical role in the clinical presentation of both recessive and dominant X-linked disorders (Additional file 1: Table S1) [152–162]. In females, mosaic XCI allows for partial compensation, as cells in which the X chromosome carrying the mutant allele is inactivated can mitigate the phenotypic impact, often resulting in milder symptoms or even asymptomatic presentations. By contrast, males, who are hemizygous for X-linked genes, generally exhibit more severe symptoms due to the complete expression of mutations on their single X chromosome. This discrepancy underscores the role of XCI in modulating disease severity [163, 164]. For neurodevelopmental disorders, where nearly 20% of X-linked genes are implicated, whether a gene is silenced or escapes XCI has a profound impact on the clinical phenotype [151]. Genes that escape inactivation in female cells can lead to overexpression, further influencing the disease course. Such mechanisms hold therapeutic potential, particularly in neural cells, where reactivating or silencing specific X-linked genes and manipulating XCI skewing may prove beneficial [147, 150, 165]. However, protein transfer (when possible) and cell selection interplay further influence the clinical manifestations in female heterozygotes [166]. Functional proteins produced by WT cells can compensate for deficits in mutant cells, while cell selection dynamics in early development —favoring either WT or mutant cells—can amplify the impact of the disease. When mutant cells gain a growth advantage, they may outcompete WT cells, worsening disease severity [166].

X-linked dominant disorders primarily affect females, as males carrying pathogenic mutations often experience embryonic lethality [32, 164, 167] (Additional file 1: Table S1). The genes implicated in several X-linked dominant disorders have been identified and can be categorized based on their phenotype in relation to the XCI pattern (see selected examples below). This classification highlights the diverse regulatory mechanisms of X-linked genes and their influence on disease pathology [35]. In some instances, the disease phenotype aligns closely with the known function of the associated gene, as seen with *OFD1* (Oral–facial-digital type I syndrome). OFD syndrome type I is marked by early male lethality and central nervous defects in 40% of cases, leading to intellectual disability, hydrocephalus, and other brain abnormalities) [168]. The *OFD1* gene encodes a protein expressed in affected tissues, with most mutations causing protein truncation and loss of function [169, 170]. However, in other cases, the connection between gene function and the observed phenotype is unclear [35]. For example, certain genes are generally expressed and play vital roles for cellular function, yet their associated disorders display highly tissue-specific symptoms. This discrepancy may arise from differences in each tissue’s capacity to handle cell damage or dysfunction when the X chromosome with the WT allele is inactivated [35].

Rett syndrome (OMIM 312750) serves as an example of a disease linked to a gene that undergoes random XCI, resulting from mutations in the gene encoding methyl-CpG-binding protein 2 (*MECP2*) [171, 172] (Additional file 1: Table S1). The loss of this essential protein predominantly affects females (1 in 10,000 female births). Nearly 900 pathogenic mutations have been identified, with half clustering in eight hotspots [172, 173] and affected females develop normally for the first 6–18 months but then regress, losing verbal and motor skills and exhibiting gait abnormalities and stereotypic hand movements [174]. While regression stabilizes over time patients require lifelong palliative care [175]. Other dominant X-linked disorders associated with genes subject to inactivation include microphthalmia with linear skin defects (MLS, OMIM 309801) [176–178]. MLS is a rare condition characterized by microphthalmia, linear skin defects, intellectual disability, seizures, and occasionally cardiac anomalies. It results from deletions or unbalanced translocations involving the Xp22.3 region, causing monosomy. Despite some phenotypic overlap with Aicardi and Goltz syndromes, MLS is now recognized as a distinct disorder [178] (Additional file 1: Table S1).

### Different approaches for treating X-linked dominant disorders

**Fig. 4:**
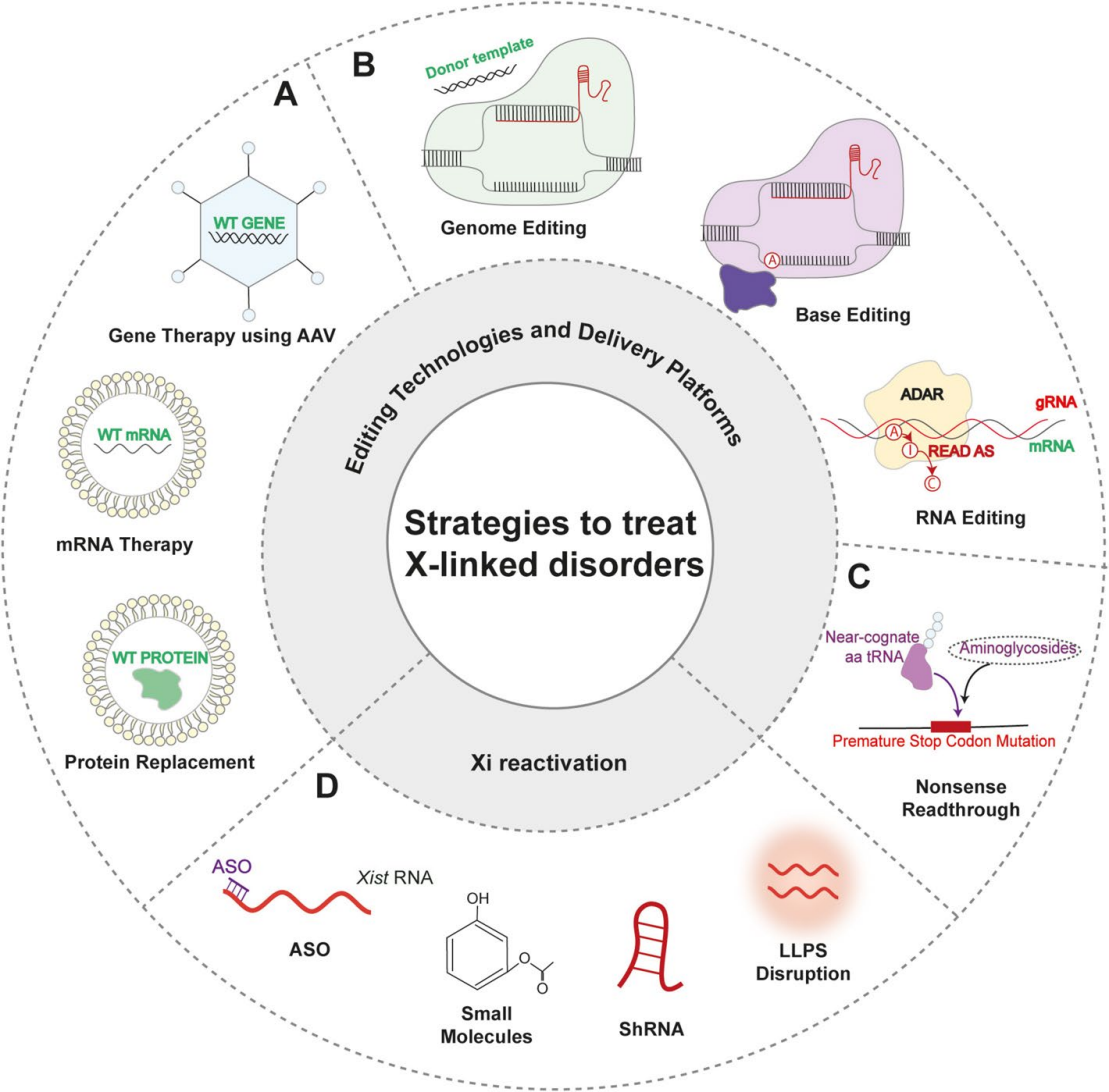
Therapeutic strategies for X-linked disorders. These methodologies include both gene function restoration and targeted editing, as well as reactivation of the inactive X chromosome. **A** Functional restoration via AAV (adeno‑associated viral)‑mediated gene therapy, mRNA‑based treatments, and protein replacement. **B** DNA and RNA editing approaches, including CRISPR‑Cas9 genome editing, base editing, and RNA editing. **C** Nonsense read‑through therapies to bypass premature stop codons. **D** Xi reactivation strategies employing antisense oligonucleotides (ASOs), small‑molecule modulators, short hairpin RNAs (shRNAs), and disruption of liquid–liquid phase separation (LLPS)

Most dominant X-linked syndromes currently lack a cure, but promising advancements are paving the way for potential therapeutic interventions. These approaches can be broadly categorized into four main groups: (i) functional gene restoration, (ii) gene-editing technologies, (iii) nonsense read-through therapies, and (iv) reactivation of the Xi (Fig. 4).

#### Functional restoration

Gene transfer, mRNA therapeutics (MRT), and protein replacement all aim to reintroduce a working gene product. In gene transfer, adeno‑associated virus (AAV) vectors deliver a synthetic copy of the defective gene [179], achieving long‑term expression but risking immune reactions (especially at high doses), inflammation, and limited brain distribution [180] (Fig. 4A). MRT encapsulates codon‑optimized, UTR‑modified mRNA (e.g., MECP2) into lipid nanoparticles to restore protein function without DNA alteration [181–184], yet is limited by innate immune sensing and blood–brain barrier penetration [185]. Protein replacement therapy directly supplies recombinant protein to bypass the genetic defect, but depends on correct post‑translational modification, precise cellular targeting, frequent dosing, and carries overexpression toxicity risks [186].

#### DNA and RNA-editing technologies

Direct correction of genetic mutations is exemplified by CRISPR–Cas9, which employs guide RNAs to introduce site‑specific DNA breaks and relies on homology‑directed repair (HDR) with an exogenous template to restore the wild‑type sequence [187] (Fig. 4B). Because HDR is inefficient in post‑mitotic neurons [188] base editors were developed to catalyze precise C → T or A → G conversions without creating double‑strand breaks [189]. These tools have reached editing efficiencies of up to 60% in murine cortex, although effective delivery and off‑target editing remain hurdles. To avoid genomic alterations entirely, RNA editing uses ADAR2 guided by programmable RNAs to deaminate adenosine into inosine—interpreted as guanosine during translation [190]—making it especially attractive for correcting G > A transitions that give rise to early stop codons [191, 192] (Fig. 4B). While this approach is inherently cell cycle–independent and effective in vitro and in certain in vivo models, current limitations include narrow mutation scope, gRNA design challenges, immunogenicity, and delivery constraints [193, 194].

#### Nonsense read-through therapies

An additional strategy is nonsense mutation read-through which aims to bypass premature stop codons in the mutated gene by promoting the insertion of near-cognate tRNA [195–197] (Fig. 4C). Approaches like the use of aminoglycosides have been tested to promote this read-through effect, but results have been inconsistent [198, 199]. Difficulties such as limited drug delivery across the blood–brain barrier (BBB), the risk of toxicity at therapeutic doses, and the lack of target specificity, which could impact other proteins, remain significant obstacles [200].

#### Reactivation of the Xi

Reactivating the Xi offers a promising strategy for heterozygous females by restoring expression from the silent, healthy gene copy (Fig. 4D). This can be achieved through small molecules, shRNA knockdown of *Xist*-interacting proteins, or ASOs targeting *Xist* lncRNA [201–203]. However, concerns remain regarding brain delivery, toxicity, broad gene reactivation, and vector-related immune-reaction risk (Fig. 4D) [116]. Targeting signaling pathways has also proven effective: inhibition of MAPK and Gsk3, along with Akt activation, promotes Xi destabilization, mimicking double-X dosage [204]. Pathways beyond MAPK and Gsk3, as for instance BMP/TGFß and Aurora kinases, which are required for proper XCI maintenance, have emerged as potential therapeutic targets [205]. Other strategies involve modulating trans-acting XCI factors (e.g., DNMT1, STC1) to alter *Xist* expression and localization [206] or using JAK/STAT inhibitors like AG490 and Jaki to restore *MeCP2* in Rett models [207].

Combinatorial approaches, such as using 5-azadC with Aurora kinase inhibitors, or SGK1 and ACVR1 inhibitors, have shown efficacy in enhancing reactivation and rescuing disease phenotypes [115, 208]. Despite progress, challenges like poor brain delivery, limited efficacy in neurons, and off-target effects persist [115, 205]. Leveraging gene-specific features—such as promoter type, gene length, and X-chromosome evolutionary strata—may aid in developing targeted reactivation strategies [201, 209]. Small molecules with BBB permeability and favorable pharmacokinetics are one of the most promising avenues to treat X-linked female biased disorders [201, 210].

### Exploring LLPS modulation as a therapeutic avenue in XCI maintenance

The following section discusses emerging ideas and hypothetical strategies, with a focus on how LLPS modulation might be harnessed in future therapeutic approaches to XCI-related disorders. Recent progress in understanding the dynamics of RNA and RBP condensates has underscored their role in maintaining cellular balance and highlighted the potential for therapeutic intervention in diseases linked to their dysfunction [211–213]. Ligands can influence condensates through thermodynamic control, a concept known as polyphasic linkage. This principle explains how compounds can modulate the balance of interactions—either enhancing or suppressing phase separation—by targeting scaffold macromolecules [214, 215]. Targeting condensates with small molecules offers a new strategy to precisely alter their molecular composition, structural organization, and behavior [216, 217]

Disrupting the internal network or promoting the dissolution of these assemblies could help restore cellular equilibrium in pathological conditions. Emerging evidence links altered condensate behavior to diseases like neurodegeneration [218]. In amyotrophic lateral sclerosis (ALS) and frontotemporal dementia, the primarily nuclear RBP TDP-43 forms pathological cytoplasmic inclusions [219]. ALS-associated mutations in the disordered regions of TDP-43 alter its LLPS properties, promoting protein aggregation [220]. Efforts to restore normal condensate behavior or eliminate aberrant TDP-43 assemblies have included strategies such as using RNA molecules, also known as aptamers [221], to prevent its inclusion formation by blocking self-interaction [222], as well as testing small molecules for their ability to modulate TDP-43 phase separation [223]. Biomolecular condensates play a significant role also in cancer by influencing gene expression through mechanisms such as super-enhancer formation and transcriptional condensate assembly, which support the overexpression of oncogenes [224, 225]. Compounds like JQ1 have shown effectiveness in disrupting condensates by dissolving super-enhancer-driven transcriptional condensates, inhibiting oncogene expression and impeding cancer cell proliferation [226]. Alternatively small molecules targeting the RBP NONO have been developed to relocate and trap this RBP in nuclear *foci* by stabilizing protein-RNA interaction, effectively reducing the expression of the androgen receptor in prostate cancer cells [227].

In the context of XCI maintenance, LLPS is likely to facilitate the recruitment and exchange of proteins with the surrounding nuclear environment, enabling the accumulation of silencing factors and thereby creating an *Xist* compartment. Manipulating phase separation represents a possible avenue to modulate *Xist* condensate function in X-linked dominant diseases, by inhibiting specific interactions or by adjusting regulatory mechanisms within these compartments. Although Xi silencing is robust and is initially believed to be permanent, partial Xi reactivation can be achieved for instance by *Xist* ablation, confirming that it is not only required for initiation of gene silencing but also for the long-term maintenance of a subset of X-linked genes [93, 120]. The possibility to selectively reactivate genes on the Xi has started to be exploited as a putative therapeutic strategy to restore the expression of missing proteins for instance in Rett syndrome [115, 119, 205, 206, 228] (see paragraph “The Xi and X-linked genetic disorders”).

**Fig. 5:**
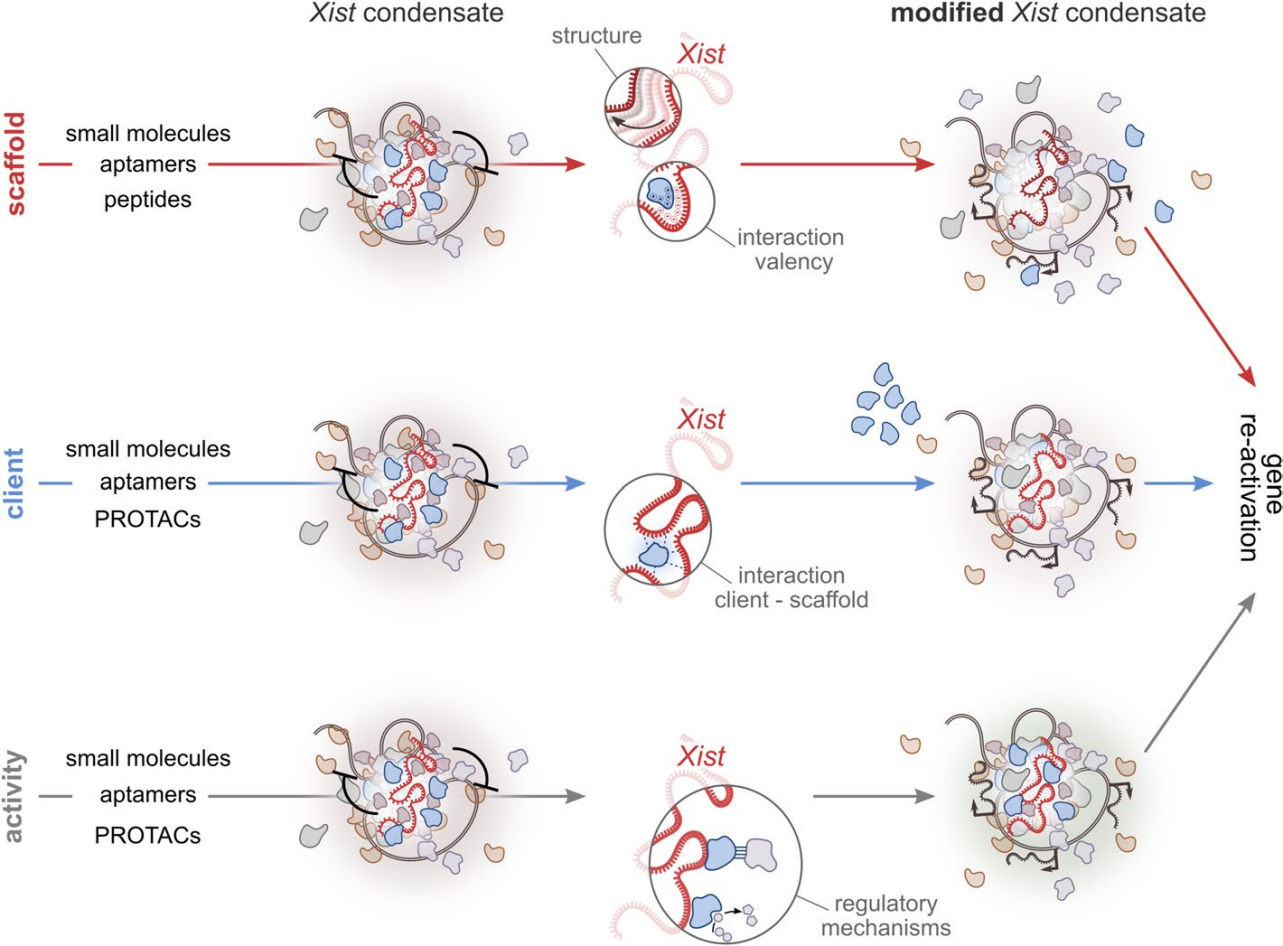
Modulation of Xist condensate for therapeutic intervention. Schematic representation of possible strategies to target the *Xist* condensate formation to reactivate gene expression from the inactive X chromosome (Xi). Top, *Targeting the scaffold* (*Xist*): Modifying the structure or interaction valency of the *Xist* RNA scaffold using small molecules, aptamers, or peptides. These changes can alter *Xist* structural properties or weaken specific molecular interactions (e.g., electrostatic interactions), leading to reduced condensate stability. Middle, *Targeting a condensate client*: Disrupting the interactions between the client molecule and the scaffold (e.g., via small molecules, aptamers, or PROTACs) can result in the exclusion or mispartitioning of specific condensate components, thereby weakening the condensate. Bottom, *Targeting condensate activity*: Modulating protein–protein interactions or enzymatic activity within the condensate can interfere with its regulatory mechanisms. This approach alters condensate function and may promote reactivation of X-linked gene expression. All strategies aim to destabilize the *Xist* condensate, enabling the reactivation of previously silenced X-linked genes

**Fig. 6:**
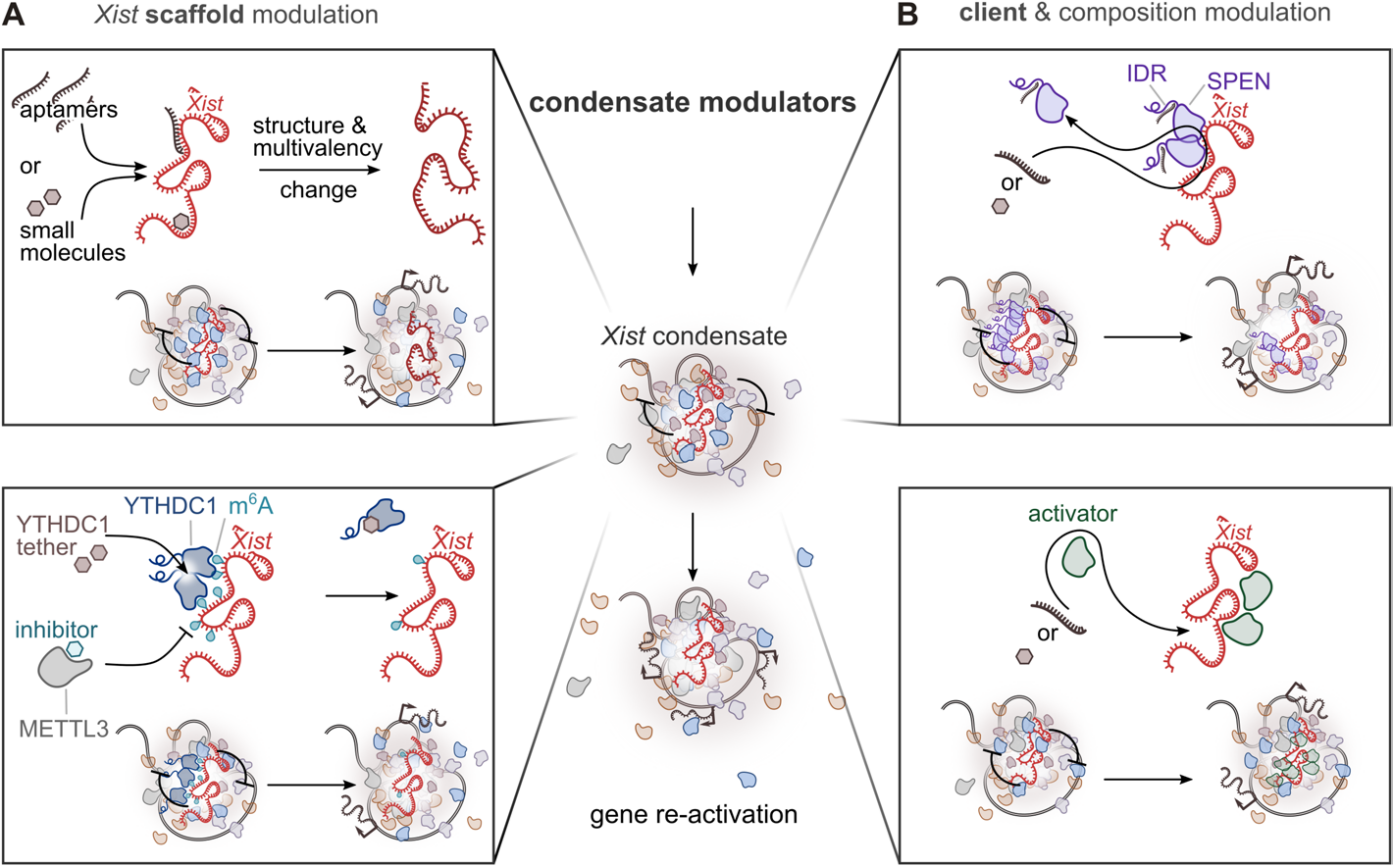
Strategies for the development of therapeutics targeting *Xist* condensates. Potential therapeutic approaches for targeting *Xist* condensates to achieve gene reactivation on the Xi. This figure represents just some of the possibilities for leveraging condensate biology as a therapeutic strategy. At the center, the general concept is depicted: targeting the *Xist* condensate to promote Xi reactivation. **A** Top square: *Xist* scaffold modulation through structural modification. Small molecules or RNA aptamers can directly bind the *Xist* RNA scaffold, altering its structure and multivalency. These modifications can disrupt RNA–protein interactions, destabilize the condensate, and promote gene reactivation. Bottom square: *Xist* scaffold modulation through m^6^A-dependent regulation. Inhibitors of the m^6^A writer METTL3 can reduce *Xist* methylation, impairing its ability to recruit m^6^A readers such as YTHDC1. Alternatively, tethering YTHDC1 directly to *Xist* can bypass the need for m^6^A, restoring condensate function and enabling precise control of gene silencing. **B** Top square: Client exclusion. Targeting client proteins recruited by *Xist*, such as SPEN (harboring an intrinsically disordered region, IDR), can disrupt essential protein interactions within the condensate, destabilizing it and enabling gene reactivation. Bottom square: *De novo* partitioning. Activators of gene expression can be selectively recruited to the condensate, promoting transcription and gene reactivation

In the context of targeting X-condensate formation for therapeutic purposes, several strategies are feasible. Depending on the desired outcome, LLPS can be modulated to either promote the partial dissolution or formation of the *Xist* condensate. Since the formation of the *Xist* condensate is essential for its silencing function, dissolving the condensate would facilitate expression reactivation, especially of genes mostly dependent on *Xist* RNA functionality, while stabilizing the condensate would maintain silencing (Fig. 5). Notably, the *Xist* compartment exhibits the characteristics of a phase-separated condensate, unlike the rest of the inactive Xi compartments [229]. This distinct property makes the *Xist*-mediated phase separation an intriguing target for therapeutic intervention. By specifically modulating the phase separation dynamics of the *Xist* condensate, it may be possible to develop highly selective therapeutic strategies aimed at reactivating silenced genes or reinforcing silencing where needed (Fig. 5). Potential approaches include (i) designing drugs that target the scaffold molecule responsible for condensate formation, i.e., *Xist*; (ii) modulating condensate composition by disrupting the recruitment or partitioning of specific client proteins; or (iii) altering the activity of specific components within the condensate itself. These possibilities are discussed in the following paragraphs (Figs. 5 and 6) and although largely hypothetical at this stage, these insights provide a conceptual framework for future studies aimed at therapeutic intervention.

### Modulate the *Xist* scaffold activity: blocking *Xist*-proteins interaction

In the formation of dynamic SMACs, *Xist* acts as a scaffold recruiting repressing proteins. Condensates form through LLPS of essential scaffold molecules, with various client molecules incorporated into the condensate via selective partitioning [230, 231] (Fig. 3C). The architectural role of *Xist* could be therapeutically targeted by modulating its multivalency, potentially inhibiting condensate formation or enhancing complex assembly, depending on the desired outcome (Figs. 5 and 6A). Modulation of the interactions facilitated by *Xist* could be achieved by influencing the folding of its repeat elements. Indeed, RNA structures can modulate the binding affinity of RNA molecules for RBPs, thereby affecting the stoichiometry of condensate components [7, 144, 232]. The A-repeat secondary structure, consisting of a single stem-loop structure repeated several times [233–235] and serves as a multimerization platform, crucial for the formation of SMACs [26, 27].

A-repeat has already been exploited in drug development through an affinity-selection mass spectrometry screen aimed at identifying small molecules that bind to *Xist* [236]. This approach identified the compound “X1,” which binds the A-repeat, disrupting *Xist* interactions with PRC2 and SPEN, inhibiting histone methylation and preventing X chromosome gene silencing [236]. The impact of X1-induced A-repeat conformational changes on *Xist* condensate formation is unknown (Fig. 6A). Targeting *Xist* requires identifying structurally complex RNA motifs for high-affinity and specific binding by drug-like molecules [237, 238]. Examples such as Branaplam [239, 240] and SMA-C5 highlight the success of small molecules targeting specific RNA structural motifs, often stabilized by protein interactions. Integrated screening strategies should be applied for the identification of small molecules targeting *Xist* RNA, including for instance (systematic evolution of ligands by exponential enrichment) SELEX or InfoRNA [241, 242]. Small-molecule ligands for *Xist* RNA must be tailored to the physicochemical features of the transcript. A positive charge is desirable to engage the negatively charged phosphate backbone, while strategically placed functional groups (e.g., hydrogen-bond donors/acceptors, π-stacking, or hydrophobic moieties) enable sequence- and structure-specific contacts within folded RNA motifs [243, 244]. Conjugating aminoglycosides with nucleobases improves RNA target specificity, as does modifying ligands to recognize stem-loop structures in oncogenic miRNA precursors [245, 246]. The rational design of small molecules or high-throughput screening for the identification of compounds binding *Xist* have to rely on the information about the RNA structure [243, 247]. Specific interactions depend on the three-dimensional structure of the target and should form specific pockets. Although several studies gave insights into the structural properties of *Xist* [39, 141, 233–235], a consensus is still lacking. Achieving a deeper understanding of *Xist* structural elements is crucial for the development of highly specific small molecules targeting its scaffolding functions.

RNA can be druggable also through the usage of oligonucleotide-based therapies, such as ASOs [248–250] or DNA/RNA aptamers. However, delivering these compounds remains highly challenging [251]. RNA aptamers, i.e., single-stranded DNA or RNA molecule that folds into distinct three-dimensional structure, binding to specific target molecules, could be engineered to bind selectively to the A-repeat region of *Xist*. By competing with or displacing *Xis*t protein partners, aptamers can alter the kinetic of interacting proteins and thereby hinder the formation and maintenance of interactions required for proper macromolecular crowding. Similarly, short peptides or small molecules that mimic key *Xist* partners could be developed. By introducing peptides that competitively inhibit the binding of *Xist* interactors, it is possible to prevent the assembly of the protein complexes that *Xist* scaffolds to form condensates. Lastly, *Xist* levels—and thus its scaffolding function—could be reduced using ribonuclease-targeting chimeras (RIBOTACs), which couple an RNA-binding ligand to a moiety that recruits and activates RNase L, triggering selective degradation of the target RNA [252].

Post-transcriptional modifications of RNA are critical regulators of its function and can influence the capacity of RNA molecules to undergo phase separation [144, 253]. *Xist* RNA is modified with m^6^A and it has been implicated in the regulation of *Xist* turnover and therefore RNA levels [254]. These sites downstream of the A-repeat have a minor effect on RNA structure, but it mediates the recruitment of YTHDC1. Importantly, YTHDC1 harbors IDRs and can drive phase separation and formation of nuclear condensate that partially overlap with other nuclear speckles [255]. The recruitment of YTHDC1 is possible to play an important role in the formation of *Xist* condensates. In this context, inhibiting m^6^A deposition could potentially diminish the phase-separation propensity of *Xist*, thereby weakening its silencing effect. Modulation of *Xist* methylation, for instance by blocking m^6^A writer METTL3/14 [256], may represent another route for condensate tuning (Fig. 6A). Notably, it has been shown that when anchored to *Xist*, YTHDC1 can rescue gene silencing following m^6^A removal [47]. Building on this, engineering YTHDC1 interactors to specifically target *Xist* condensates—such as by conjugating them to nucleic acid recognition elements designed to bind *Xist* [257]—or developing METTL3/14 inhibitors with enhanced specificity for *Xist*-associated methylation sites, could provide innovative strategies to modulate condensate dynamics and X-linked gene silencing (Fig. 6A). However, recent findings suggest that disrupting METTL3 can enhance *Xist*-mediated silencing [254], a result that contrasts with previous studies reporting an impairment of silencing upon interference with the m^6^A pathway [47, 258]. The precise role of m^6^A modification in *Xist*-mediated silencing should be clarified in the context of *Xist* condensates and for pursuing therapeutic strategies targeting this pathway. In addition, given the broad role of METTL3/14 in regulating m^6^A modification across numerous transcripts, global inhibition may lead to unintended effects on other cellular RNAs.

Regions of *Xist* beyond the A-repeat also exhibit scaffold activity, promoting the recruitment and local enrichment of various proteins to form *Xist* compartments. The E-repeat, in particular, attracts proteins such as PTBP1, MATR3, TDP-43, and CELF1, which are known to undergo phase separation and form assemblies (Fig. 4B) [28, 259, 260]. These multivalent interactions between RBPs and RNA likely play a crucial role in triggering condensate formation, resulting in the formation of the Xi compartment, which contributes to Xi [261]. In the absence of the E-repeat, silencing begins correctly but fails to be sustained over time [28]. Given the interest in gene-specific XCR, targeting the E-repeat, in addition to the A-repeat, could represent an effective strategy to disrupt *Xist* condensates and subsequently reverse X-linked gene silencing. However, it remains unclear whether the A- and E-repeats act in concert to form a unified condensate structure or if they contribute to separate yet interconnected assemblies. Similarly, the B-repeat has now been shown to influence HNRNPK condensates, playing a key role in silencing spreading [30]. Therefore also this region may hold potential for therapeutic modulation of *Xist* condensates.

### Modulating client partitioning in condensates: approaches for altering the composition of *Xist* condensates

Another strategy for *Xist* reactivation involves modulating the composition of *Xist* condensates by disrupting the interaction of factors (i.e., *Xist* clients) essential for *Xist*-mediated silencing (Fig. 5). For example, molecules that bind to the IDRs of a protein can influence its intermolecular interactions within the condensate (Fig. 6B). Different RBPs undergoing LLPS, as for instance CELF1, remain enriched in the Xi compartment—through its protein interactome—suggesting that these proteins may also play a role in shaping the *Xist* compartment even after differentiation [28]. It is unclear whether SPEN spatially remains associated within these compartments. However, homotypic multivalent interactions mediated by SPEN IDRs are essential for the formation of *Xist-*mediated assemblies, targeting these regions could significantly alter SPEN crowding and, consequently, reduce gene silencing. RNA aptamers could be engineered to target the SPEN IDR, thereby inhibiting its phase separation and neutralizing its silencing activity (Fig. 6B). RNA aptamers could be engineered—using computational tools as demonstrated in the case of aptamers targeting TDP-43 aggregation [221]—to bind *Xist*-associated proteins and control their localization within the *Xist* compartment. Saturation concentration can be adjusted by small molecules binding directly to proteins, altering their chemical properties and influencing condensate dynamics [223, 262, 263]. Small molecules have also been identified that bind to protein IDRs, enhancing the therapeutic potential of these regions [264]. For instance planar aromatic compounds, such as mitoxantrone, disrupt ALS-associated RBPs in stress granules [265], while karyopherin-β2 inhibits FUS phase separation by stabilizing interactions and preventing LLPS [266].

While disrupting client protein partitioning within *Xist* condensates may not be sufficient to remove repressive marks or induce gene reactivation on its own, it could act synergistically with chromatin-targeting strategies to enhance their efficacy (see “Different approaches for treating X-linked dominant disorders” section for more details).

SPEN and other client molecules within *Xist*-mediated assemblies could be targeted and redirected into “non-functional” condensates. For example, small molecules targeting NONO have been shown to induce its assembly into nuclear *puncta*, effectively reducing androgen receptor expression in prostate cancer cells [227].

On the one hand, removing repressive factors from *Xist* assemblies may promote the reactivation of X-linked genes; on the other hand, recruiting an activator presents an alternative approach that could be implemented concurrently (Fig. 6B). Avrainvillamide can restore the nuclear localization of NPM1 protein [267], this is a proof of principle that small molecules can selectively interact with a protein and modify its localization in a targeted manner. The loss of *Xist* RNA in somatic cells has been shown to facilitate the reappearance of certain activating factors. Indeed, *Xist* not only recruits repressive complexes but also actively repels BRG1–SWI/SNF [268] and cohesins [203]. Importantly, BRG1 potentiates XCR after treatment with a DNA methylation inhibitor (5’-azacytidine) and a topoisomerase 2b inhibitor [268]; therefore, recruiting BRG1 and promoting the BRG1–*Xist* interaction could enhance the selectivity of Xi reactivation (Fig. 6B).

In the context of therapies targeting phase-separated condensates, valuable insights can be drawn from the development of antivirals [212]. Strategies have been developed to promote the dissolution of viral condensates to disrupt their biological functions. The phase separation of the SARS-CoV-2 nucleocapsid (NC) is linked to the inactivation of innate antiviral immunity [269]. A peptide targeting the dimerization domain was screened to disrupt the LLPS of SARS-CoV-2 NC, demonstrating [270] its ability to inhibit viral replication and restore innate antiviral immunity.

Targeting components of the *Xist* condensate for degradation represents a promising strategy to interfere with gene silencing and promote X-linked gene reactivation. One potential approach involves the use of PROteolysis-Targeting Chimeras (PROTACs), heterobifunctional small molecules designed to induce for instance targeted protein degradation by recruiting E3 ubiquitin ligases to specific proteins of interest [271, 272]. PROTACs have not only progressed to late-stage clinical trials for cancer therapy but have also shown promise in treating neurodegenerative diseases [273] by targeting proteins with critical roles in neurological conditions, such as TDP-43, alpha-synuclein, and Tau [274–276]. To target *Xist* condensate components, the choice of a nuclear-localized E3 ligase, such as DCAF16, could be crucial for ensuring effective degradation within the nuclear environment [277]. Furthermore, the properties of phase-separated environments may enhance the effectiveness of PROTACs. The addition of a nuclear IDR to a PROTAC has been shown to significantly improve its ability to degrade nuclear targets [277, 278]. These findings highlight the potential of leveraging PROTAC technology to disrupt *Xist* condensates and achieve XCR.

### Affecting the activity of *Xist*-condensate components

A strategy to modulate the *Xist* condensate is by tuning the behavior of individual proteins that reside within the *Xist*-driven compartments (or SMACs) (Fig. 5). Current research on condensate-modifying therapeutics has revealed several avenues for manipulating dynamic interaction networks in biomolecular assemblies [279] without necessarily disrupting the entire condensate [211]. For instance, the silencing factor SPEN is recruited by *Xist* to initiate repression, but if SPEN activity can be dampened post-development—i.e., by blocking its interaction partners—it might partially release silenced genes. In this context, bifunctional small molecules, also known as “molecular glues,” could be particularly useful. These molecules engage multiple targets to modulate protein proximity, alter interaction networks, and inhibit specific protein–protein interactions [280].

Alter post-translational modifications (PTMs) on key condensate proteins could be used as a strategy to modify function. Studies of phase separation have shown that PTMs such as phosphorylation or ubiquitination can dramatically influence condensates formation and stability [281–284]. Hyperphosphorylation of RNA polymerase II large subunit alters its affinity from transcription condensate to those specialized in RNA splicing [284]. Translating these insights to *Xist* compartments means strategically modifying proteins essential for *Xist*-based silencing—thereby switching the local environment toward reactivation.

One promising approach to modifying condensate function is to target enzymes that reside within them [285]. Molecules can be designed to target enzymes partitioned within the *Xist* condensate. *Xist* orchestrates the inactivation of one of the two X chromosomes by recruiting multiple enzymes responsible for DNA hypermethylation and histone deacetylation. Small molecules could be designed to preferentially partition within the *Xist* compartment. This approach could enhance the efficiency of therapeutic agents and minimize off-target effects by concentrating activity within the condensate microenvironment.

Compounds that modulate phase separation represent a tool for modifying condensate behavior, material properties, and therefore function. For instance, lipoamide was recently shown to alter SG assembly via a redox regulatory pathway [286]. 4,4’-dianilino-1,1’-binaphthyl-5,5’-disulfonic acid (bis-ANS) have been shown to modulate the LLPS of TDP43 IDR. At elevated concentrations, these molecules disassemble liquid droplets via a reversible phase transition driven by electrostatic forces [223]. Alternatively, the DisCo (Disassembly of Condensates) technique demonstrates a proof of principle for disrupting biomolecular condensates. By using chemical-induced dimerization to recruit a ligand near the condensate-forming region of a scaffold protein, this method induces condensate dissociation. While the precise mechanism remains unclear, DisCo highlights the potential for targeted disruption of condensates as a therapeutic strategy [287]. However, in the context of *Xist* condensates, a complete dissolution or change in material of the compartments is unlikely to be the desired outcome of a therapeutic strategy, as excessive reactivation of X-linked genes could lead to negative effects that outweigh the intended upregulation of the target gene.

By applying these principles to the *Xist* condensate, it may be possible to dismantle or remodel the silencing environment (Figs. 5 and 6). Critically, the *Xist*-induced protein gradients that expand silencing across the chromosome rely on proper condensate assembly and maintenance. Small molecules capable of altering local protein concentration or compartment coalescence could promote selected genes reawakening.

### Challenges and perspectives for LLPS-based Xi reactivation strategies

Intervening in LLPS dynamics underlying *Xist*-mediated silencing offers a compelling opportunity to semi-selectively reactivate genes on Xi. However, several critical considerations must be addressed to transform this concept into therapies.

First, although SMACs on the Xi have been hypothesized to form via LLPS—supported by the finding that SPEN accumulation in SMACs depends on IDRs—it remains uncertain whether these assemblies fully exhibit hallmarks of LLPS [26, 29]. While some concentration-dependent assemblies indeed arise from LLPS, this is not the only mechanism by which they can form [31, 128, 288]. As such, in-depth characterization of the biophysical and chemical properties of *Xist*-induced compartments is essential, including refined assays to determine their composition and to probe the three-dimensional structure of *Xist* within these condensates. A robust understanding of how *Xist* drives molecular crowding in these domains is pivotal for targeted interventions. From a therapeutic perspective, reactivation of the Xi would likely need to occur in somatic cells, as proper XCI is essential for early embryonic development [289]. Therefore, as postulated for other Xi reactivation strategies [290], targeting *Xist* condensates would ideally be applied in a post-XCI context. Supporting this view, experimental studies in mice have demonstrated that post-natal Xi reactivation in somatic cells can be well tolerated without significant morbidity [121, 291]. Apparently distinct *Xist*-protein condensates have been described, each associated with specific *Xist* repeats (A-repeat, B-repeat, or E-repeat) and protein factors. However, it remains unclear whether these condensates represent parallel structures or reflect functionally distinct stages of XCI progression. If these condensates indeed mediate different phases of XCI, selectively targeting specific structures could help identify the optimal therapeutic window. For instance, E-repeat–dependent protein assemblies have been shown to maintain gene silencing independently of *Xist* in mouse cells [28], suggesting that targeting proteins recruited via the E-repeat might specifically influence the maintenance of established XCI in somatic cells. Several of these proteins have also been implicated in sustaining gene repression on the Xi in adult human cells [146]. Thus, it is plausible that *Xist* condensate components remain functionally relevant beyond the initiation phase, potentially making the Xi amenable to targeted interventions even in post-XCI somatic contexts. We hypothesize that targeting *Xist* condensates during the initiation of XCI could disrupt the early recruitment of repressive complexes and hinder the proper establishment of gene silencing. In contrast, targeting condensates during the maintenance phase of XCI might interfere with the continued interactions required to sustain silencing, potentially facilitating expression of previously silent genes but not achieving whole Xi reactivation. It is crucial to investigate whether condensates involved in XCI initiation differ structurally or functionally from those maintaining the silent state, and highlight the importance of studying condensate dynamics at both stages to inform therapeutic strategies.

Second, appropriate screening platforms are needed to evaluate chemical libraries or rationally designed molecules that could disrupt or modulate *Xist* condensates, thereby reactivating specific X-linked genes. Such strategies might include high-throughput screening, phenotypic assays, and computational methods, but one must keep in mind the species-specific differences in *Xist* activity [238]. Indeed, molecules effective in mouse models may not always translate directly to human *XIST*. Additionally, identifying cellular and mouse models that faithfully recapitulate Xi silencing will be critical for demonstrating both efficacy and safety [290]. While the involvement of *Xist* condensates in XCI has been primarily characterized in mouse models, it remains to be determined whether similar condensate-based mechanisms operate in human cells. Future studies will be essential to determine whether condensate formation is a conserved hallmark of *XIST* activity in the human system. Nevertheless, insights from mouse models may help define whether targeting such assemblies could be effective in X-linked disorders.

Third, even if effective Xi reactivation is achieved for a target locus (e.g., *MECP2* in Rett syndrome), there is a risk of unwanted re-expression of other X-linked genes. For example, depletion of *Xist* in mouse models triggers reactivation of over ten additional genes [291], underscoring the need for careful titration of reactivation levels. Nonetheless, in certain disease contexts—particularly Rett syndrome—therapeutic benefit of restoring *MECP2* likely outweighs potential side effects from partial reactivation of additional *loci*.

Lastly, the challenge of drug delivery remains a major hurdle. Therapeutic approaches relying on Xi reactivation will necessarily depend on the specific organs or tissues affected by the disorder. In this regard, strategies aimed at reactivating Xi should ideally include selective targeting to minimize unnecessary exposure of unaffected tissues and organs. Because Rett syndrome manifests as a neurodevelopmental disorder, therapeutic agents must effectively cross the BBB and achieve sufficient intracellular concentrations in the relevant neural tissues. Moreover, any therapeutic intervention would likely require repeated administration to sustain its beneficial effects, posing challenges related to treatment frequency and long-term feasibility.

These requirements add another layer of design complexity, as carriers or formulations that enable brain-specific targeting may be necessary for clinical success.

## Conclusions

XCI plays a critical role in gene dosage parity and regulating X-linked gene expression in female mammals [4–6, 292]. During early development, XCI silences one of the two X chromosomes in somatic cells, orchestrated by the *Xist* RNA, which recruits repressive proteins to form a compact heterochromatin structure [2, 7]. The stability of XCI is maintained through chromatin modifications and nuclear compartmentalization, ensuring that the silenced state is kept [37].

Phenotypic variability in X-linked disorders is shaped by XCI mosaicism in females, with cells expressing either the WT or mutant X chromosome [34, 36]. In contrast, in males, mutations on the single X chromosome are often lethal, emphasizing the importance of XCI for understanding X-linked diseases [35]. Studies on XCR have provided valuable insights into repressive chromatin erasure and epigenetic reprogramming, offering new therapeutic opportunities for reactivating the WT X chromosome and correcting X-linked mutations [36].

LLPS has emerged as a key mechanism in XCI, with *Xist* RNA functioning as a scaffold that concentrates RBPs and repressive complexes into specialized condensates (SMACs). These structures optimize molecular interactions to enforce gene silencing across the X chromosome while maintaining specificity. Biomolecular condensates have emerged as critical players in various diseases—including cancer and neurodegeneration—making them highly relevant for the development of novel therapeutic strategies [211, 218, 226, 285, 293]. Understanding the formation and regulation of *Xist* specialized—possibly phase-separated—condensates opens new avenues for therapeutic reactivation of the inactive X chromosome, by targeting mechanisms that have so far remained unexplored. What are the advantages to target macromolecular crowding in XCI?

(i) The *Xist* condensates are localized, potentially reducing off-target effects versus broad-acting drugs (e.g., global DNA demethylation agents). Furthermore, studies have found that small molecules have the possibility to be selectively recruited within a certain condensate [217], as demonstrated by cisplatin and other anticancer drugs enrichment in transcriptional condensates [226]. These findings suggest the potential to design small molecules that not only exhibit high specificity for their target proteins but also accumulate preferentially within the intended condensates, thereby enhancing their therapeutic effectiveness [226].
(ii) Condensates can be highly dynamic with constant exchange of molecules with the surrounding nuclear environment [231], disassembling or modifying these structures enabling a finely tuned approach to X-linked gene reactivation rather than binary on–off strategy.
(iii) XCI involves numerous steps and a large repertoire of molecular complexes. Combining drugs that target distinct aspects of the *Xist* condensate could enhance reactivation, potentially in synergy with established reactivation strategies [290].

From a translational perspective, modulating *Xist* condensates offers promising avenues for X-linked disease therapies, such as Rett syndrome. However, their dynamic, multicomponent nature may require strategies beyond traditional single-target drug approaches. Off-target risks also exist, as many condensate components are shared across cell types. Designing precision molecules—such as bifunctional compounds that bind *Xist* and alter condensate stability—may overcome these limitations. For example a bifunctional JQ1–polyamide compound reactivated the silenced *FXN* gene in Friedreich’s ataxia, illustrating the potential of precision-targeted therapeutics [294].

Moving forward, cross-disciplinary efforts will be essential—combining RNA biology, phase-separation, and drug discovery—to translate these concepts into targeted therapies for X-linked diseases.

## Supplementary Information

Additional file 1: Table S1. X-Linked Dominant Diseases as Potential Targets for Xi Reactivation. This table lists ten X-linked dominant disorders that may represent potential therapeutic targets for reactivation of the inactive X chromosome (Xi), providing disease names and relevant context.

## Data Availability

No datasets were generated or analysed during the current study.

## References

[1] Cerase A, Pintacuda G, Tattermusch A, Avner P. Xist localization and function: new insights from multiple levels. Genome Biol. 2015;16:166. 26282267 10.1186/s13059-015-0733-y PMC4539689

[2] Heard E. Recent advances in X-chromosome inactivation. Curr Opin Cell Biol. 2004;16:247–55. 15145348 10.1016/j.ceb.2004.03.005

[3] Pessia E, Engelstädter J, Marais GAB. The evolution of X chromosome inactivation in mammals: the demise of Ohno’s hypothesis? Cell Mol Life Sci. 2013;71:1383–94. 24173285 10.1007/s00018-013-1499-6 PMC11113734

[4] Disteche CM. Dosage compensation of the sex chromosomes. Annu Rev Genet. 2012;46:537–60. 22974302 10.1146/annurev-genet-110711-155454 PMC3767307

[5] Pintacuda G, Cerase A. X Inactivation lessons from differentiating mouse embryonic stem cells. Stem Cell Rev Rep. 2015;11:699–705. 26198263 10.1007/s12015-015-9597-5 PMC4561061

[6] Quinn JJ, Chang HY. Unique features of long non-coding RNA biogenesis and function. Nat Rev Genet. 2016;17:47–62. 26666209 10.1038/nrg.2015.10

[7] Pintacuda G, Young AN, Cerase A. Function by structure: spotlights on Xist long non-coding RNA. Front Mol Biosci. 2017;4:90. 29302591 10.3389/fmolb.2017.00090 PMC5742192

[8] Anguera MC, Ma W, Clift D, Namekawa S, Kelleher RJ, Lee JT. Tsx produces a long noncoding RNA and has general functions in the germline, stem cells, and brain. PLoS Genet. 2011;7:e1002248. 21912526 10.1371/journal.pgen.1002248 PMC3164691

[9] Ogawa Y, Lee JT. Xite, X-inactivation intergenic transcription elements that regulate the probability of choice. Mol Cell. 2003;11:731–43. 12667455 10.1016/s1097-2765(03)00063-7

[10] Stavropoulos N, Rowntree RK, Lee JT. Identification of developmentally specific enhancers for Tsix in the regulation of X chromosome inactivation. Mol Cell Biol. 2005;25:2757–69. 15767680 10.1128/MCB.25.7.2757-2769.2005 PMC1061649

[11] Chureau C, Chantalat S, Romito A, Galvani A, Duret L, Avner P, Rougeulle C. Ftx is a non-coding RNA which affects Xist expression and chromatin structure within the X-inactivation center region. Hum Mol Genet. 2011;20:705–18. 21118898 10.1093/hmg/ddq516

[12] Sun Z, Zhu M, Lv P, Cheng L, Wang Q, Tian P, Yan Z, Wen B. The long noncoding RNA Lncenc1 maintains naive states of mouse ESCs by promoting the glycolysis pathway. Stem Cell Rep. 2018;11:741–55. 10.1016/j.stemcr.2018.08.001 PMC6135739 30174313

[13] Tian D, Sun S, Lee JT. The long noncoding RNA, Jpx, is a molecular switch for X chromosome inactivation. Cell. 2010;143:390–403. 21029862 10.1016/j.cell.2010.09.049 PMC2994261

[14] Furlan G, Gutierrez Hernandez N, Huret C, Galupa R, van Bemmel JG, Romito A, Heard E, Morey C, Rougeulle C. The Ftx noncoding locus controls X chromosome inactivation independently of its RNA products. Mol Cell. 2018;70:462-472.e8. 29706539 10.1016/j.molcel.2018.03.024

[15] Nora EP, Lajoie BR, Schulz EG, et al. Spatial partitioning of the regulatory landscape of the X-inactivation centre. Nature. 2012;485:381–5. 22495304 10.1038/nature11049 PMC3555144

[16] Navarro P, Chambers I, Karwacki-Neisius V, Chureau C, Morey C, Rougeulle C, Avner P. Molecular coupling of Xist regulation and pluripotency. Science. 2008;321:1693–5. 18802003 10.1126/science.1160952

[17] Minkovsky A, Barakat TS, Sellami N, Chin MH, Gunhanlar N, Gribnau J, Plath K. The pluripotency factor-bound intron 1 of Xist is dispensable for X chromosome inactivation and reactivation in vitro and *in vivo*. Cell Rep. 2013;3:905–18. 23523354 10.1016/j.celrep.2013.02.018 PMC3615097

[18] Nesterova TB, Senner CE, Schneider J, Alcayna-Stevens T, Tattermusch A, Hemberger M, Brockdorff N. Pluripotency factor binding and Tsix expression act synergistically to repress Xist in undifferentiated embryonic stem cells. Epigenetics Chromatin. 2011;4:17. 21982142 10.1186/1756-8935-4-17 PMC3197471

[19] Genolet O, Monaco AA, Dunkel I, Boettcher M, Schulz EG. Identification of X-chromosomal genes that drive sex differences in embryonic stem cells through a hierarchical CRISPR screening approach. Genome Biol. 2021;22:110. 33863351 10.1186/s13059-021-02321-2 PMC8051100

[20] Cerase A, Young AN, Ruiz NB, et al. Chd8 regulates X chromosome inactivation in mouse through fine-tuning control of Xist expression. Commun Biol. 2021;4:1–13. 33859315 10.1038/s42003-021-01945-1 PMC8050208

[21] Keniry A, Gearing LJ, Jansz N, et al. Setdb1-mediated H3K9 methylation is enriched on the inactive X and plays a role in its epigenetic silencing. Epigenetics Chromatin. 2016;9:16. 27195021 10.1186/s13072-016-0064-6 PMC4870784

[22] Galupa R, Heard E. X-chromosome inactivation: a crossroads between chromosome architecture and gene regulation. Annu Rev Genet. 2018;52:535–66. 30256677 10.1146/annurev-genet-120116-024611

[23] Dossin F, Pinheiro I, Żylicz JJ, et al. SPEN integrates transcriptional and epigenetic control of X-inactivation. Nature. 2020;578:455–60. 32025035 10.1038/s41586-020-1974-9 PMC7035112

[24] Raposo AC, Casanova M, Gendrel AV, da Rocha ST. The tandem repeat modules of Xist lncRNA: a swiss army knife for the control of X-chromosome inactivation. Biochem Soc Trans. 2021;49:2549–60. 34882219 10.1042/BST20210253 PMC8786293

[25] Hasegawa Y, Brockdorff N, Kawano S, Tsutui K, Tsutui K, Nakagawa S. The matrix protein hnRNP U is required for chromosomal localization of Xist RNA. Dev Cell. 2010;19:469–76. 20833368 10.1016/j.devcel.2010.08.006

[26] Markaki Y, Gan Chong J, Wang Y, et al. Xist nucleates local protein gradients to propagate silencing across the X chromosome. Cell. 2021;184:6174-6192.e32. 34813726 10.1016/j.cell.2021.10.022 PMC8671326

[27] Cerase A, Armaos A, Neumayer C, Avner P, Guttman M, Tartaglia GG. Phase separation drives X-chromosome inactivation: a hypothesis. Nat Struct Mol Biol. 2019;26:331–4. 31061525 10.1038/s41594-019-0223-0

[28] Pandya-Jones A, Markaki Y, Serizay J, et al. A protein assembly mediates Xist localization and gene silencing. Nature. 2020;587:145–51. 32908311 10.1038/s41586-020-2703-0 PMC7644664

[29] Jachowicz JW, Strehle M, Banerjee AK, Blanco MR, Thai J, Guttman M. Xist spatially amplifies SHARP/SPEN recruitment to balance chromosome-wide silencing and specificity to the X chromosome. Nat Struct Mol Biol. 2022;29:239–49. 35301492 10.1038/s41594-022-00739-1 PMC8969943

[30] Ding M, Wang D, Chen H, et al. A biophysical basis for the spreading behavior and limited diffusion of Xist. Cell. 2025. 10.1016/j.cell.2024.12.004. 39824183 10.1016/j.cell.2024.12.004 PMC11863002

[31] A P, Weber SC. Evidence for and against liquid-liquid phase separation in the nucleus. Noncoding RNA. 2019;5:50. 31683819 10.3390/ncrna5040050 PMC6958436

[32] Zacco E, Broglia L, Kurihara M, et al. RNA: the unsuspected conductor in the orchestra of macromolecular crowding. Chem Rev. 2024;124:4734–77. 38579177 10.1021/acs.chemrev.3c00575 PMC11046439

[33] Oh T, Cho S, Yoo C, Yeo W, Oh J, Seo M. Polymerization-induced microphase separation of a polymerization mixture into nanostructured block polymer materials. Prog Polym Sci. 2023;145:101738.

[34] Chaligné R, Heard E. X-chromosome inactivation in development and cancer. FEBS Lett. 2014;588:2514–22. 24937141 10.1016/j.febslet.2014.06.023

[35] Franco B, Ballabio A. X-inactivation and human disease: X-linked dominant male-lethal disorders. Curr Opin Genet Dev. 2006;16:254–9. 16650755 10.1016/j.gde.2006.04.012

[36] Talon I, Janiszewski A, Chappell J, Vanheer L, Pasque V. Recent advances in understanding the reversal of gene silencing during X chromosome reactivation. Front Cell Dev Biol. 2019;7:169. 31552244 10.3389/fcell.2019.00169 PMC6733891

[37] Mak W, Nesterova TB, de Napoles M, Appanah R, Yamanaka S, Otte AP, Brockdorff N. Reactivation of the paternal X chromosome in early mouse embryos. Science. 2004;303:666–9. 14752160 10.1126/science.1092674

[38] Okamoto I, Patrat C, Thépot D, et al. Eutherian mammals use diverse strategies to initiate X-chromosome inactivation during development. Nature. 2011;472:370–4. 21471966 10.1038/nature09872

[39] Wutz A, Rasmussen TP, Jaenisch R. Chromosomal silencing and localization are mediated by different domains of Xist RNA. Nat Genet. 2002;30:167–74. 11780141 10.1038/ng820

[40] Borensztein M, Syx L, Ancelin K, et al. Xist-dependent imprinted X inactivation and the early developmental consequences of its failure. Nat Struct Mol Biol. 2017;24:226–33. 28134930 10.1038/nsmb.3365 PMC5337400

[41] Alfeghaly C, Castel G, Cazottes E, et al. XIST dampens X chromosome activity in a SPEN-dependent manner during early human development. Nat Struct Mol Biol. 2024;31:1589–600. 38834912 10.1038/s41594-024-01325-3 PMC11479943

[42] McHugh CA, Chen CK, Chow A, et al. The Xist lncRNA interacts directly with SHARP to silence transcription through HDAC3. Nature. 2015;521:232–6. 25915022 10.1038/nature14443 PMC4516396

[43] Trigiante G, Blanes Ruiz N, Cerase A. Emerging roles of repetitive and repeat-containing RNA in nuclear and chromatin organization and gene expression. Front Cell Dev Biol. 2021;9:735527. 34722514 10.3389/fcell.2021.735527 PMC8552494

[44] Monfort A, Di Minin G, Postlmayr A, Freimann R, Arieti F, Thore S, Wutz A. Identification of Spen as a crucial factor for Xist function through forward genetic screening in haploid embryonic stem cells. Cell Rep. 2015;12:554–61. 26190100 10.1016/j.celrep.2015.06.067 PMC4530576

[45] Moindrot B, Cerase A, Coker H, Masui O, Grijzenhout A, Pintacuda G, Schermelleh L, Nesterova TB, Brockdorff N. A pooled shRNA screen identifies Rbm15, Spen, and Wtap as factors required for Xist RNA-mediated silencing. Cell Rep. 2015;12:562–72. 26190105 10.1016/j.celrep.2015.06.053 PMC4534822

[46] Robert-Finestra T, Tan BF, Mira-Bontenbal H, et al. SPEN is required for Xist upregulation during initiation of X chromosome inactivation. Nat Commun. 2021;12:7000. 34853312 10.1038/s41467-021-27294-5 PMC8636516

[47] Patil DP, Chen CK, Pickering BF, Chow A, Jackson C, Guttman M, Jaffrey SR. m(6)A RNA methylation promotes XIST-mediated transcriptional repression. Nature. 2016;537:369–73. 27602518 10.1038/nature19342 PMC5509218

[48] da Rocha ST, Heard E. Novel players in X inactivation: insights into Xist-mediated gene silencing and chromosome conformation. Nat Struct Mol Biol. 2017;24:197–204. 28257137 10.1038/nsmb.3370

[49] da Rocha ST, Boeva V, Escamilla-Del-Arenal M, et al. Jarid2 Is implicated in the initial Xist-induced targeting of PRC2 to the inactive X chromosome. Mol Cell. 2014;53:301–16. 24462204 10.1016/j.molcel.2014.01.002

[50] Chu C, Zhang QC, da Rocha ST, Flynn RA, Bharadwaj M, Calabrese JM, Magnuson T, Heard E, Chang HY. Systematic discovery of Xist RNA binding proteins. Cell. 2015;161:404–16. 25843628 10.1016/j.cell.2015.03.025 PMC4425988

[51] Navarro-Cobos MJ, Brown CJ. Recruitment of chromatin remodelers by XIST B-repeat region is variably dependent on HNRNPK. Hum Mol Genet. 2025;34:229–38. 39588742 10.1093/hmg/ddae173 PMC11792242

[52] Żylicz JJ, Bousard A, Žumer K, et al. The implication of early chromatin changes in X chromosome inactivation. Cell. 2019;176:182-197.e23. 30595450 10.1016/j.cell.2018.11.041 PMC6333919

[53] Simon MD, Pinter SF, Fang R, Sarma K, Rutenberg-Schoenberg M, Bowman SK, Kesner BA, Maier VK, Kingston RE, Lee JT. High-resolution Xist binding maps reveal two-step spreading during X-chromosome inactivation. Nature. 2013;504:465–9. 24162848 10.1038/nature12719 PMC3904790

[54] Schertzer MD, Braceros KCA, Starmer J, et al. lncRNA-induced spread of Polycomb controlled by genome architecture, RNA abundance, and CpG island DNA. Mol Cell. 2019;75:523-537.e10. 31256989 10.1016/j.molcel.2019.05.028 PMC6688959

[55] Pinter SF, Sadreyev RI, Yildirim E, Jeon Y, Ohsumi TK, Borowsky M, Lee JT. Spreading of X chromosome inactivation via a hierarchy of defined Polycomb stations. Genome Res. 2012;22:1864–76. 22948768 10.1101/gr.133751.111 PMC3460182

[56] Almeida M, Pintacuda G, Masui O, et al. PCGF3/5-PRC1 initiates Polycomb recruitment in X chromosome inactivation. Science. 2017;356:1081–4. 28596365 10.1126/science.aal2512 PMC6522364

[57] Engreitz JM, Pandya-Jones A, McDonel P, et al. The Xist lncRNA exploits three-dimensional genome architecture to spread across the X chromosome. Science. 2013;341:1237973. 23828888 10.1126/science.1237973 PMC3778663

[58] Bousard A, Raposo AC, Żylicz JJ, et al. The role of Xist-mediated Polycomb recruitment in the initiation of X-chromosome inactivation. EMBO Rep. 2019;20:e48019. 31456285 10.15252/embr.201948019 PMC6776897

[59] Özeş AR, Pulliam N, Ertosun MG, Yılmaz Ö, Tang J, Çopuroğlu E, Matei D, Özeş ON, Nephew KP. Protein kinase A-mediated phosphorylation regulates STAT3 activation and oncogenic EZH2 activity. Oncogene. 2018;37:3589–600. 29576612 10.1038/s41388-018-0218-z PMC6023775

[60] Schwartz YB, Kahn TG, Nix DA, Li XY, Bourgon R, Biggin M, Pirrotta V. Genome-wide analysis of Polycomb targets in Drosophila melanogaster. Nat Genet. 2006;38:700–5. 16732288 10.1038/ng1817

[61] Boyer LA, Plath K, Zeitlinger J, et al. Polycomb complexes repress developmental regulators in murine embryonic stem cells. Nature. 2006;441:349–53. 16625203 10.1038/nature04733

[62] Bowness JS, Nesterova TB, Wei G, Rodermund L, Almeida M, Coker H, Carter EJ, Kadaster A, Brockdorff N. Xist-mediated silencing requires additive functions of SPEN and Polycomb together with differentiation-dependent recruitment of SmcHD1. Cell Rep. 2022;39:110830. 35584662 10.1016/j.celrep.2022.110830 PMC9637994

[63] Wang CY, Jégu T, Chu HP, Oh HJ, Lee JT. SMCHD1 merges chromosome compartments and assists formation of super-structures on the inactive X. Cell. 2018;174:406-421.e25. 29887375 10.1016/j.cell.2018.05.007 PMC6475921

[64] Gendrel AV, Apedaile A, Coker H, et al. Smchd1-dependent and -independent pathways determine developmental dynamics of CpG island methylation on the inactive X chromosome. Dev Cell. 2012;23:265–79. 22841499 10.1016/j.devcel.2012.06.011 PMC3437444

[65] Jansz N, Nesterova T, Keniry A, et al. Smchd1 targeting to the inactive X is dependent on the Xist-HnrnpK-PRC1 pathway. Cell Rep. 2018;25:1912-1923.e9. 30428357 10.1016/j.celrep.2018.10.044

[66] Sakakibara Y, Nagao K, Blewitt M, Sasaki H, Obuse C, Sado T. Role of SmcHD1 in establishment of epigenetic states required for the maintenance of the X-inactivated state in mice. Development. 2018;145:dev166462. 30126901 10.1242/dev.166462

[67] Gdula MR, Nesterova TB, Pintacuda G, et al. The non-canonical SMC protein SmcHD1 antagonises TAD formation and compartmentalisation on the inactive X chromosome. Nat Commun. 2019;10:30. 30604745 10.1038/s41467-018-07907-2 PMC6318279

[68] Wang CY, Colognori D, Sunwoo H, Wang D, Lee JT. PRC1 collaborates with SMCHD1 to fold the X-chromosome and spread Xist RNA between chromosome compartments. Nat Commun. 2019;10:2950. 31270318 10.1038/s41467-019-10755-3 PMC6610634

[69] Blewitt ME, Gendrel AV, Pang Z, et al. SmcHD1, containing a structural-maintenance-of-chromosomes hinge domain, has a critical role in X inactivation. Nat Genet. 2008;40:663–9. 18425126 10.1038/ng.142

[70] Sunwoo H, Colognori D, Froberg JE, Jeon Y, Lee JT. Repeat E anchors Xist RNA to the inactive X chromosomal compartment through CDKN1A-interacting protein (CIZ1). Proc Natl Acad Sci U S A. 2017;114:10654–9. 28923964 10.1073/pnas.1711206114 PMC5635913

[71] Ridings-Figueroa R, Stewart ER, Nesterova TB, et al. The nuclear matrix protein CIZ1 facilitates localization of Xist RNA to the inactive X-chromosome territory. Genes Dev. 2017;31:876–88. 28546514 10.1101/gad.295907.117 PMC5458755

[72] Makhlouf M, Ouimette JF, Oldfield A, Navarro P, Neuillet D, Rougeulle C. A prominent and conserved role for YY1 in Xist transcriptional activation. Nat Commun. 2014;5:4878. 25209548 10.1038/ncomms5878 PMC4172967

[73] Jacobson EC, Pandya-Jones A, Plath K. A lifelong duty: how *Xist* maintains the inactive X chromosome. Curr Opin Genet Dev. 2022;75:101927. 35717799 10.1016/j.gde.2022.101927 PMC9472561

[74] Young AN, Perlas E, Ruiz-Blanes N, Hierholzer A, Pomella N, Martin-Martin B, Liverziani A, Jachowicz JW, Giannakouros T, Cerase A. Deletion of LBR N-terminal domains recapitulates Pelger-Huet anomaly phenotypes in mouse without disrupting X chromosome inactivation. Commun Biol. 2021;4:1–8. 33846535 10.1038/s42003-021-01944-2 PMC8041748

[75] Jeon Y, Lee JT. YY1 tethers Xist RNA to the inactive X nucleation center. Cell. 2011;146:119–33. 21729784 10.1016/j.cell.2011.06.026 PMC3150513

[76] Chapman AG, Cotton AM, Kelsey AD, Brown CJ. Differentially methylated CpG island within human XIST mediates alternative P2 transcription and YY1 binding. BMC Genet. 2014;15:89. 25200388 10.1186/s12863-014-0089-4 PMC4363909

[77] Chen CK, Blanco M, Jackson C, Aznauryan E, Ollikainen N, Surka C, Chow A, Cerase A, McDonel P, Guttman M. Xist recruits the X chromosome to the nuclear lamina to enable chromosome-wide silencing. Science. 2016;354:468–72. 27492478 10.1126/science.aae0047

[78] Loda A, Heard E. Xist RNA in action: past, present, and future. PLoS Genet. 2019;15:e1008333. 31537017 10.1371/journal.pgen.1008333 PMC6752956

[79] Pollex T, Heard E. Nuclear positioning and pairing of X-chromosome inactivation centers are not primary determinants during initiation of random X-inactivation. Nat Genet. 2019;51:285–95. 30643252 10.1038/s41588-018-0305-7 PMC7617203

[80] Sakaguchi T, Hasegawa Y, Brockdorff N, Tsutsui K, Tsutsui KM, Sado T, Nakagawa S. Control of chromosomal localization of Xist by hnRNP U family molecules. Dev Cell. 2016;39:11–2. 27728779 10.1016/j.devcel.2016.09.022

[81] Agostini F, Cirillo D, Bolognesi B, Tartaglia GG. X-inactivation: quantitative predictions of protein interactions in the Xist network. Nucleic Acids Res. 2013;41:e31. 23093590 10.1093/nar/gks968 PMC3592426

[82] Yamada N, Hasegawa Y, Yue M, Hamada T, Nakagawa S, Ogawa Y. Xist Exon 7 contributes to the stable localization of Xist RNA on the inactive X-chromosome. PLoS Genet. 2015;11:e1005430. 26244333 10.1371/journal.pgen.1005430 PMC4526699

[83] Monfort A, Wutz A. The B-side of Xist. F1000Res. 2020;9:F1000 Faculty Rev-55.32047616

[84] Lv Q, Yuan L, Song Y, Sui T, Li Z, Lai L. D-repeat in the XIST gene is required for X chromosome inactivation. RNA Biol. 2016;13:172–6. 26786668 10.1080/15476286.2015.1137420 PMC4829313

[85] Teller K, Illner D, Thamm S, Casas-Delucchi CS, Versteeg R, Indemans M, Cremer T, Cremer M. A top-down analysis of Xa- and Xi-territories reveals differences of higher order structure at ≥ 20 Mb genomic length scales. Nucleus. 2011;2:465–77. 21970989 10.4161/nucl.2.5.17862

[86] Collombet S, Ranisavljevic N, Nagano T, et al. Parental-to-embryo switch of chromosome organization in early embryogenesis. Nature. 2020;580:142–6. 32238933 10.1038/s41586-020-2125-z

[87] Petersen GB, Therelsen AJ. Number of nucleoli in female and male human cells in tissue culture. Exp Cell Res. 1962;28:590–2. 13942751 10.1016/0014-4827(62)90264-1

[88] Bourgeois CA, Laquerriere F, Hemon D, Hubert J, Bouteille M. New data on the in-situ position of the inactive X chromosome in the interphase nucleus of human fibroblasts. Hum Genet. 1985;69:122–9. 3972413 10.1007/BF00293281

[89] Barr ML, Bertram EG. A morphological distinction between neurones of the male and female, and the behaviour of the nucleolar satellite during accelerated nucleoprotein synthesis. Nature. 1949;163:676. 18120749 10.1038/163676a0

[90] Barton DE, David FN, Merrington M. The positions of the sex chromosomes in the human cell in mitosis. Ann Hum Genet. 1964;28:123–8. 14227999 10.1111/j.1469-1809.1964.tb00467.x

[91] Belmont AS, Bignone F, Ts’o PO. The relative intranuclear positions of Barr bodies in XXX non-transformed human fibroblasts. Exp Cell Res. 1986;165:165–79. 3709685 10.1016/0014-4827(86)90541-0

[92] Rego A, Sinclair PB, Tao W, Kireev I, Belmont AS. The facultative heterochromatin of the inactive X chromosome has a distinctive condensed ultrastructure. J Cell Sci. 2008;121:1119–27. 18334550 10.1242/jcs.026104

[93] Zhang LF, Huynh KD, Lee JT. Perinucleolar targeting of the inactive X during S phase: evidence for a role in the maintenance of silencing. Cell. 2007;129:693–706. 17512404 10.1016/j.cell.2007.03.036

[94] Dyer KA, Canfield TK, Gartler SM. Molecular cytological differentiation of active from inactive X domains in interphase: implications for X chromosome inactivation. Cytogenet Cell Genet. 1989;50:116–20. 2776476 10.1159/000132736

[95] Eils R, Dietzel S, Bertin E, Schröck E, Speicher MR, Ried T, Robert-Nicoud M, Cremer C, Cremer T. Three-dimensional reconstruction of painted human interphase chromosomes: active and inactive X chromosome territories have similar volumes but differ in shape and surface structure. J Cell Biol. 1996;135:1427–40. 8978813 10.1083/jcb.135.6.1427 PMC2133958

[96] Rao SSP, Huntley MH, Durand NC, et al. A 3D map of the human genome at kilobase resolution reveals principles of chromatin looping. Cell. 2014;159:1665–80. 25497547 10.1016/j.cell.2014.11.021 PMC5635824

[97] Giorgetti L, Lajoie BR, Carter AC, et al. Structural organization of the inactive X chromosome in the mouse. Nature. 2016;535:575–9. 27437574 10.1038/nature18589 PMC5443622

[98] Darrow EM, Huntley MH, Dudchenko O, et al. Deletion of DXZ4 on the human inactive X chromosome alters higher-order genome architecture. Proc Natl Acad Sci U S A. 2016;113:E4504-4512. 27432957 10.1073/pnas.1609643113 PMC4978254

[99] Deng X, Ma W, Ramani V, et al. Bipartite structure of the inactive mouse X chromosome. Genome Biol. 2015;16:152. 26248554 10.1186/s13059-015-0728-8 PMC4539712

[100] Martitz A, Schulz EG. Spatial orchestration of the genome: topological reorganisation during X-chromosome inactivation. Curr Opin Genet Dev. 2024;86:102198. 38663040 10.1016/j.gde.2024.102198

[101] Miura H, Poonperm R, Takahashi S, Hiratani I. Practical analysis of Hi-C data: generating A/B compartment profiles. Methods Mol Biol. 2018;1861:221–45. 30218370 10.1007/978-1-4939-8766-5_16

[102] Chaumeil J, Baccon PL, Wutz A, Heard E. A novel role for Xist RNA in the formation of a repressive nuclear compartment into which genes are recruited when silenced. Genes Dev. 2006;20:2223–37. 16912274 10.1101/gad.380906 PMC1553206

[103] Bauer M, Vidal E, Zorita E, Üresin N, Pinter SF, Filion GJ, Payer B. Chromosome compartments on the inactive X guide TAD formation independently of transcription during X-reactivation. Nat Commun. 2021;12:3499. 34108480 10.1038/s41467-021-23610-1 PMC8190187

[104] Bowness JS, Almeida M, Nesterova TB, Brockdorff N. YY1 binding is a gene-intrinsic barrier to Xist-mediated gene silencing. EMBO Rep. 2024;25:2258–77. 38654121 10.1038/s44319-024-00136-3 PMC11094009

[105] Hernández-Muñoz I, Lund AH, van der Stoop P, Boutsma E, Muijrers I, Verhoeven E, Nusinow DA, Panning B, Marahrens Y, van Lohuizen M. Stable X chromosome inactivation involves the PRC1 Polycomb complex and requires histone MACROH2A1 and the CULLIN3/SPOP ubiquitin E3 ligase. Proc Natl Acad Sci U S A. 2005;102:7635–40. 15897469 10.1073/pnas.0408918102 PMC1140410

[106] Zhuang M, Calabrese MF, Liu J, et al. Structures of SPOP-substrate complexes: insights into molecular architectures of BTB-Cul3 ubiquitin ligases. Mol Cell. 2009;36:39–50. 19818708 10.1016/j.molcel.2009.09.022 PMC2847577

[107] Dobrinić P, Szczurek AT, Klose RJ. PRC1 drives Polycomb-mediated gene repression by controlling transcription initiation and burst frequency. Nat Struct Mol Biol. 2021;28:811–24. 34608337 10.1038/s41594-021-00661-y PMC7612713

[108] Tanasijevic B, Rasmussen TP. X chromosome inactivation and differentiation occur readily in ES cells doubly-deficient for macroH2A1 and macroH2A2. PLoS One. 2011;6:e21512. 21738686 10.1371/journal.pone.0021512 PMC3127949

[109] Pehrson JR, Changolkar LN, Costanzi C, Leu NA. Mice without macroH2A histone variants. Mol Cell Biol. 2014;34:4523–33. 25312643 10.1128/MCB.00794-14 PMC4248737

[110] Changolkar LN, Costanzi C, Leu NA, Chen D, McLaughlin KJ, Pehrson JR. Developmental changes in histone macroH2A1-mediated gene regulation. Mol Cell Biol. 2007;27:2758–64. 17242180 10.1128/MCB.02334-06 PMC1899912

[111] Weber M, Hellmann I, Stadler MB, Ramos L, Pääbo S, Rebhan M, Schübeler D. Distribution, silencing potential and evolutionary impact of promoter DNA methylation in the human genome. Nat Genet. 2007;39:457–66. 17334365 10.1038/ng1990

[112] Sado T, Fenner MH, Tan SS, Tam P, Shioda T, Li E. X inactivation in the mouse embryo deficient for Dnmt1: distinct effect of hypomethylation on imprinted and random X inactivation. Dev Biol. 2000;225:294–303. 10985851 10.1006/dbio.2000.9823

[113] Norris DP, Brockdorff N, Rastan S. Methylation status of CpG-rich islands on active and inactive mouse X chromosomes. Mamm Genome. 1991;1:78–83. 1799791 10.1007/BF02443782

[114] Li R, Goswami U, King M, Chen J, Cesario TC, Rentzepis PM. In situ detection of live-to-dead bacteria ratio after inactivation by means of synchronous fluorescence and PCA. Proc Natl Acad Sci U S A. 2018;115:668–73. 29311322 10.1073/pnas.1716514115 PMC5789938

[115] Lessing D, Dial TO, Wei C, et al. A high-throughput small molecule screen identifies synergism between DNA methylation and Aurora kinase pathways for X reactivation. Proc Natl Acad Sci U S A. 2016;113:14366–71. 28182563 10.1073/pnas.1617597113 PMC5167172

[116] Minajigi A, Froberg JE, Wei C, et al. A comprehensive Xist interactome reveals cohesin repulsion and an RNA-directed chromosome conformation. Science. 2015;349:aab2276. 26089354 10.1126/science.aab2276 PMC4845908

[117] Minkovsky A, Sahakyan A, Rankin-Gee E, Bonora G, Patel S, Plath K. The Mbd1-Atf7ip-Setdb1 pathway contributes to the maintenance of X chromosome inactivation. Epigenetics Chromatin. 2014;7:12. 25028596 10.1186/1756-8935-7-12 PMC4099106

[118] Wang Y, Zhong Y, Zhou Y, Tanaseichuk O, Li Z, Zhao JC. Identification of a Xist silencing domain by Tiling CRISPR. Sci Rep. 2019;9:2408. 30787302 10.1038/s41598-018-36750-0 PMC6382781

[119] Carrette LLG, Wang CY, Wei C, Press W, Ma W, Kelleher RJ, Lee JT. A mixed modality approach towards Xi reactivation for Rett syndrome and other X-linked disorders. Proc Natl Acad Sci U S A. 2018;115:E668–75. 29282321 10.1073/pnas.1715124115 PMC5789928

[120] Yildirim E, Kirby JE, Brown DE, Mercier FE, Sadreyev RI, Scadden DT, Lee JT. Xist RNA is a potent suppressor of hematologic cancer in mice. Cell. 2013;152:727–42. 23415223 10.1016/j.cell.2013.01.034 PMC3875356

[121] Yang L, Yildirim E, Kirby JE, Press W, Lee JT. Widespread organ tolerance to Xist loss and X reactivation except under chronic stress in the gut. Proc Natl Acad Sci. 2020;117:4262–72. 32041873 10.1073/pnas.1917203117 PMC7049159

[122] Wutz A, Jaenisch R. A shift from reversible to irreversible X inactivation is triggered during ES cell differentiation. Mol Cell. 2000;5:695–705. 10882105 10.1016/s1097-2765(00)80248-8

[123] Brangwynne CP, Tompa P, Pappu RV. Polymer physics of intracellular phase transitions. Nature Phys. 2015;11:899–904.

[124] Harmon TS, Holehouse AS, Rosen MK, Pappu RV. Intrinsically disordered linkers determine the interplay between phase separation and gelation in multivalent proteins. Elife. 2017;6:e30294. 29091028 10.7554/eLife.30294 PMC5703641

[125] Li P, Banjade S, Cheng HC, et al. Phase transitions in the assembly of multivalent signalling proteins. Nature. 2012;483:336–40. 22398450 10.1038/nature10879 PMC3343696

[126] Banani SF, Lee HO, Hyman AA, Rosen MK. Biomolecular condensates: organizers of cellular biochemistry. Nat Rev Mol Cell Biol. 2017;18:285–98. 28225081 10.1038/nrm.2017.7 PMC7434221

[127] Sabari BR, Dall’Agnese A, Young RA. Biomolecular condensates in the nucleus. Trends Biochem Sci. 2020;45:961–77. 32684431 10.1016/j.tibs.2020.06.007 PMC7572565

[128] Lyon AS, Peeples WB, Rosen MK. A framework for understanding the functions of biomolecular condensates across scales. Nat Rev Mol Cell Biol. 2021;22:215–35. 33169001 10.1038/s41580-020-00303-z PMC8574987

[129] Vandelli A, Cid Samper F, Torrent Burgas M, Sanchez de Groot N, Gaetano Tartaglia G. Interplay between disordered regions in RNAs and proteins modulates interactions within stress granules and processing bodies. J Mol Biol. 2021;434:167159. 34274326 10.1016/j.jmb.2021.167159

[130] Banani SF, Rice AM, Peeples WB, Lin Y, Jain S, Parker R, Rosen MK. Compositional control of phase-separated cellular bodies. Cell. 2016;166:651–63. 27374333 10.1016/j.cell.2016.06.010 PMC4967043

[131] Monti M, Fiorentino J, Miltiadis-Vrachnos D, Bini G, Cotrufo T, Sanchez de Groot N, Armaos A, Tartaglia GG. catGRANULE 2.0: accurate predictions of liquid-liquid phase separating proteins at single amino acid resolution. Genome Biol. 2025;26:33. 39979996 10.1186/s13059-025-03497-7 PMC11843755

[132] Sunwoo H, Wu JY, Lee JT. The Xist RNA-PRC2 complex at 20-nm resolution reveals a low Xist stoichiometry and suggests a hit-and-run mechanism in mouse cells. Proc Natl Acad Sci U S A. 2015;112:E4216-4225. 26195790 10.1073/pnas.1503690112 PMC4534268

[133] Cerase A, Smeets D, Tang YA, et al. Spatial separation of Xist RNA and polycomb proteins revealed by superresolution microscopy. Proc Natl Acad Sci U S A. 2014;111:2235–40. 24469834 10.1073/pnas.1312951111 PMC3926064

[134] Cerase A, Calabrese JM, Tartaglia GG. Phase separation drives X-chromosome inactivation. Nat Struct Mol Biol. 2022;29:183–5. 35301494 10.1038/s41594-021-00697-0

[135] Cerase A, Tartaglia GG. Long non-coding RNA-polycomb intimate rendezvous. Open Biol. 2020;10:200126. 32898472 10.1098/rsob.200126 PMC7536065

[136] Delli Ponti R, Marti S, Armaos A, Tartaglia GG. A high-throughput approach to profile RNA structure. Nucleic Acids Res. 2017;45:e35. 27899588 10.1093/nar/gkw1094 PMC5389523

[137] Yamazaki T, Souquere S, Chujo T, Kobelke S, Chong YS, Fox AH, Bond CS, Nakagawa S, Pierron G, Hirose T. Functional domains of NEAT1 architectural lncRNA induce paraspeckle assembly through phase separation. Mol Cell. 2018;70:1038-1053.e7. 29932899 10.1016/j.molcel.2018.05.019

[138] Plath K, Fang J, Mlynarczyk-Evans SK, Cao R, Worringer KA, Wang H, de la Cruz CC, Otte AP, Panning B, Zhang Y. Role of histone H3 lysine 27 methylation in X inactivation. Science. 2003;300:131–5. 12649488 10.1126/science.1084274

[139] Markaki Y, Smeets D, Fiedler S, Schmid VJ, Schermelleh L, Cremer T, Cremer M. The potential of 3D-FISH and super-resolution structured illumination microscopy for studies of 3D nuclear architecture: 3D structured illumination microscopy of defined chromosomal structures visualized by 3D (immuno)-FISH opens new perspectives for studies of nuclear architecture. BioEssays. 2012;34:412–26. 22508100 10.1002/bies.201100176

[140] Rodermund L, Coker H, Oldenkamp R, Wei G, Bowness J, Rajkumar B, Nesterova T, Susano Pinto DM, Schermelleh L, Brockdorff N. Time-resolved structured illumination microscopy reveals key principles of Xist RNA spreading. Science. 2021;372:eabe7500. 34112668 10.1126/science.abe7500

[141] Jones AN, Sattler M. Challenges and perspectives for structural biology of lncRNAs-the example of the Xist lncRNA A-repeats. J Mol Cell Biol. 2019;11:845–59. 31336384 10.1093/jmcb/mjz086 PMC6917512

[142] Cirillo D, Blanco M, Armaos A, Buness A, Avner P, Guttman M, Cerase A, Tartaglia GG. Quantitative predictions of protein interactions with long noncoding RNAs. Nat Methods. 2017;14:5–6. 10.1038/nmeth.4100 28032625

[143] Michaels TCT, Wutz A. Phase separation paints Xi with Xist. Cell Res. 2025:1–2. 10.1038/s41422-025-01116-5. 10.1038/s41422-025-01116-5 PMC12408801 40229556

[144] Roden C, Gladfelter AS. RNA contributions to the form and function of biomolecular condensates. Nat Rev Mol Cell Biol. 2021;22:183–95. 32632317 10.1038/s41580-020-0264-6 PMC7785677

[145] Brockdorff N, Ashworth A, Kay GF, Cooper P, Smith S, McCabe VM, Norris DP, Penny GD, Patel D, Rastan S. Conservation of position and exclusive expression of mouse Xist from the inactive X chromosome. Nature. 1991;351:329–31. 2034279 10.1038/351329a0

[146] Yu B, Qi Y, Li R, Shi Q, Satpathy AT, Chang HY. B cell-specific XIST complex enforces X-inactivation and restrains atypical B cells. Cell. 2021;184:1790-1803.e17. 33735607 10.1016/j.cell.2021.02.015 PMC9196326

[147] Santiwatana S, Mahachoklertwattana P, Limwongse C, Khlairit P, Pongratanakul S, Roothumnong E, Prangphan K, Choubtum L, Songdej D, Poomthavorn P. Skewed X chromosome inactivation in girls and female adolescents with autoimmune thyroid disease. Clin Endocrinol (Oxf). 2018;89:863–9. 30229980 10.1111/cen.13857

[148] Chabchoub G, Uz E, Maalej A, Mustafa CA, Rebai A, Mnif M, Bahloul Z, Farid NR, Ozcelik T, Ayadi H. Analysis of skewed X-chromosome inactivation in females with rheumatoid arthritis and autoimmune thyroid diseases. Arthritis Res Ther. 2009;11:R106. 19589151 10.1186/ar2759 PMC2745787

[149] Orstavik KH. X chromosome inactivation in clinical practice. Hum Genet. 2009;126:363–73. 19396465 10.1007/s00439-009-0670-5

[150] Plenge RM, Stevenson RA, Lubs HA, Schwartz CE, Willard HF. Skewed X-chromosome inactivation is a common feature of X-linked mental retardation disorders. Am J Hum Genet. 2002;71:168–73. 12068376 10.1086/341123 PMC384975

[151] Fieremans N, Bauters M, Belet S, Verbeeck J, Jansen AC, Seneca S, Roelens F, De Baere E, Marynen P, Froyen G. De novo MECP2 duplications in two females with intellectual disability and unfavorable complete skewed X-inactivation. Hum Genet. 2014;133:1359–67. 25037250 10.1007/s00439-014-1469-6

[152] Dimitrov DS. Therapeutic proteins. Methods Mol Biol. 2012;899:1–26. 22735943 10.1007/978-1-61779-921-1_1 PMC6988726

[153] Powers S, Miranda C, Dennys-Rivers C, et al. Rett syndrome gene therapy improves survival and ameliorates behavioral phenotypes in MeCP2 null (S5.1002). Neurology. 2019;92(S51):002.

[154] Sinnett SE, Boyle E, Lyons C, Gray SJ. Engineered microRNA-based regulatory element permits safe high-dose miniMECP2 gene therapy in Rett mice. Brain. 2021;144:3005–19. 33950254 10.1093/brain/awab182 PMC8783608

[155] Gadalla KKE, Bailey MES, Spike RC, et al. Improved survival and reduced phenotypic severity following AAV9/MECP2 gene transfer to neonatal and juvenile male Mecp2 knockout mice. Mol Ther. 2013;21:18–30. 23011033 10.1038/mt.2012.200 PMC3536818

[156] Sinnamon JR, Kim SY, Fisk JR, Song Z, Nakai H, Jeng S, McWeeney SK, Mandel G. *In Vivo* repair of a protein underlying a neurological disorder by programmable RNA editing. Cell Rep. 2020;32:107878. 32668243 10.1016/j.celrep.2020.107878 PMC7449137

[157] Pitcher MR, Herrera JA, Buffington SA, Kochukov MY, Merritt JK, Fisher AR, Schanen NC, Costa-Mattioli M, Neul JL. Rett syndrome like phenotypes in the R255X Mecp2 mutant mouse are rescued by MECP2 transgene. Hum Mol Genet. 2015;24:2662–72. 25634563 10.1093/hmg/ddv030 PMC4383870

[158] Popescu AC, Sidorova E, Zhang G, Eubanks JH. Aminoglycoside-mediated partial suppression of MECP2 nonsense mutations responsible for Rett syndrome in vitro. J Neurosci Res. 2010;88:2316–24. 20623622 10.1002/jnr.22409

[159] Fazzari M, Frasca A, Bifari F, Landsberger N. Aminoglycoside drugs induce efficient read-through of CDKL5 nonsense mutations, slightly restoring its kinase activity. RNA Biol. 2019;16:1414–23. 31232219 10.1080/15476286.2019.1632633 PMC6779400

[160] Voronin G, Narasimhan J, Gittens J, et al. Preclinical studies of gene replacement therapy for CDKL5 deficiency disorder. Mol Ther. 2024;32:3331–45. 39033321 10.1016/j.ymthe.2024.07.012 PMC11489525

[161] Medici G, Tassinari M, Galvani G, et al. Expression of a secretable, cell-penetrating CDKL5 protein enhances the efficacy of gene therapy for CDKL5 deficiency disorder. Neurotherapeutics. 2022;19:1886–904. 36109452 10.1007/s13311-022-01295-8 PMC9723029

[162] Halmai JANM, Deng P, Gonzalez CE, et al. Artificial escape from XCI by DNA methylation editing of the CDKL5 gene. Nucleic Acids Res. 2020;48:2372–87. 31925439 10.1093/nar/gkz1214 PMC7049732

[163] Wang Y, Lu L, Zhang D, Tan Y, Li D, He F, Jiao X, Yang M, Hejtmancik JF, Liu X. A novel mutation of the RPGR gene in a Chinese X-linked retinitis pigmentosa family and possible involvement of X-chromosome inactivation. Eye (Lond). 2021;35:1688–96. 32839555 10.1038/s41433-020-01150-0 PMC8169654

[164] Tsang SH, Sharma T. X-linked retinitis pigmentosa. Adv Exp Med Biol. 2018;1085:31–5. 30578481 10.1007/978-3-319-95046-4_8

[165] Brand BA, Blesson AE, Smith-Hicks CL. The impact of X-chromosome inactivation on phenotypic expression of X-linked neurodevelopmental disorders. Brain Sci. 2021;11:904. 34356138 10.3390/brainsci11070904 PMC8305405

[166] Migeon BR. X-linked diseases: susceptible females. Genet Med. 2020;22:1156–74. 32284538 10.1038/s41436-020-0779-4 PMC7332419

[167] Giovenino C, Trajkova S, Pavinato L, et al. Skewed X-chromosome inactivation in unsolved neurodevelopmental disease cases can guide re-evaluation for X-linked genes. Eur J Hum Genet. 2023;31:1228–36. 36879111 10.1038/s41431-023-01324-w PMC10620389

[168] Odent S, Le Marec B, Toutain A, David A, Vigneron J, Tréguier C, Jouan H, Milon J, Fryns JP, Verloes A. Central nervous system malformations and early end-stage renal disease in oro-facio-digital syndrome type I: a review. Am J Med Genet. 1998;75:389–94. 9482645

[169] Ferrante MI, Giorgio G, Feather SA, et al. Identification of the gene for oral-facial-digital type I syndrome. Am J Hum Genet. 2001;68:569–76. 11179005 10.1086/318802 PMC1274470

[170] de Conciliis L, Marchitiello A, Wapenaar MC, et al. Characterization of Cxorf5 (71–7A), a novel human cDNA mapping to Xp22 and encoding a protein containing coiled-coil alpha-helical domains. Genomics. 1998;51:243–50. 9722947 10.1006/geno.1998.5348

[171] Amir RE, Van den Veyver IB, Wan M, Tran CQ, Francke U, Zoghbi HY. Rett syndrome is caused by mutations in X-linked MECP2, encoding methyl-CpG-binding protein 2. Nat Genet. 1999;23:185–8. 10508514 10.1038/13810

[172] Wan M, Lee SS, Zhang X, et al. Rett syndrome and beyond: recurrent spontaneous and familial MECP2 mutations at CpG hotspots. Am J Hum Genet. 1999;65:1520–9. 10577905 10.1086/302690 PMC1288362

[173] Ehrhart F, Jacobsen A, Rigau M, et al. A catalogue of 863 Rett-syndrome-causing MECP2 mutations and lessons learned from data integration. Sci Data. 2021;8:10. 33452270 10.1038/s41597-020-00794-7 PMC7810705

[174] Neul JL, Kaufmann WE, Glaze DG, et al. Rett syndrome: revised diagnostic criteria and nomenclature. Ann Neurol. 2010;68:944–50. 21154482 10.1002/ana.22124 PMC3058521

[175] Dolce A, Ben-Zeev B, Naidu S, Kossoff EH. Rett syndrome and epilepsy: an update for child neurologists. Pediatr Neurol. 2013;48:337–45. 23583050 10.1016/j.pediatrneurol.2012.11.001

[176] Doege TC, Thuline HC, Priest JH, Norby DE, Bryant JS. Studies of a family with the oral-facial-digital syndrome. N Engl J Med. 1964;271:1073–8. 14210999 10.1056/NEJM196411192712101

[177] Wettke-Schäfer R, Kantner G. X-linked dominant inherited diseases with lethality in hemizygous males. Hum Genet. 1983;64:1–23. 6873941 10.1007/BF00289472

[178] Morleo M, Pramparo T, Perone L, et al. Microphthalmia with linear skin defects (MLS) syndrome: clinical, cytogenetic, and molecular characterization of 11 cases. Am J Med Genet A. 2005;137:190–8. 16059943 10.1002/ajmg.a.30864

[179] Wang D, Tai PWL, Gao G. Adeno-associated virus vector as a platform for gene therapy delivery. Nat Rev Drug Discov. 2019;18:358–78. 30710128 10.1038/s41573-019-0012-9 PMC6927556

[180] Shirley JL, de Jong YP, Terhorst C, Herzog RW. Immune responses to viral gene therapy vectors. Mol Ther. 2020;28:709–22. 31968213 10.1016/j.ymthe.2020.01.001 PMC7054714

[181] Rungta RL, Choi HB, Lin PJ, Ko RW, Ashby D, Nair J, Manoharan M, Cullis PR, Macvicar BA. Lipid nanoparticle delivery of siRNA to silence neuronal gene expression in the brain. Mol Ther Nucleic Acids. 2013;2:e136. 24301867 10.1038/mtna.2013.65 PMC3889191

[182] Pardi N, Hogan MJ, Porter FW, Weissman D. mRNA vaccines — a new era in vaccinology. Nat Rev Drug Discov. 2018;17:261–79. 29326426 10.1038/nrd.2017.243 PMC5906799

[183] Buschmann MD, Carrasco MJ, Alishetty S, Paige M, Alameh MG, Weissman D. Nanomaterial delivery systems for mRNA vaccines. Vaccines. 2021;9:65. 33478109 10.3390/vaccines9010065 PMC7836001

[184] Kanasty R, Dorkin JR, Vegas A, Anderson D. Delivery materials for siRNA therapeutics. Nature Mater. 2013;12:967–77. 24150415 10.1038/nmat3765

[185] Tosi G, Musumeci T, Ruozi B, Carbone C, Belletti D, Pignatello R, Vandelli MA, Puglisi G. The “fate” of polymeric and lipid nanoparticles for brain delivery and targeting: strategies and mechanism of blood–brain barrier crossing and trafficking into the central nervous system. J Drug Deliv Sci Technol. 2016;32:66–76.

[186] Bellini E, Pavesi G, Barbiero I, et al. MeCP2 post-translational modifications: a mechanism to control its involvement in synaptic plasticity and homeostasis? Front Cell Neurosci. 2014. 10.3389/fncel.2014.00236. 25165434 10.3389/fncel.2014.00236 PMC4131190

[187] Ran FA, Hsu PD, Wright J, Agarwala V, Scott DA, Zhang F. Genome engineering using the CRISPR-Cas9 system. Nat Protoc. 2013;8:2281–308. 24157548 10.1038/nprot.2013.143 PMC3969860

[188] Croci S, Carriero ML, Capitani K, et al. High rate of HDR in gene editing of p. (Thr158Met) MECP2 mutational hotspot. Eur J Hum Genet. 2020;28:1231–42. 32332872 10.1038/s41431-020-0624-x PMC7609331

[189] Porto EM, Komor AC, Slaymaker IM, Yeo GW. Base editing: advances and therapeutic opportunities. Nat Rev Drug Discov. 2020;19:839–59. 33077937 10.1038/s41573-020-0084-6 PMC7721651

[190] Komor AC, Kim YB, Packer MS, Zuris JA, Liu DR. Programmable editing of a target base in genomic DNA without double-stranded DNA cleavage. Nature. 2016;533:420–4. 27096365 10.1038/nature17946 PMC4873371

[191] Fukuda M, Umeno H, Nose K, Nishitarumizu A, Noguchi R, Nakagawa H. Construction of a guide-RNA for site-directed RNA mutagenesis utilising intracellular A-to-I RNA editing. Sci Rep. 2017;7:41478. 28148949 10.1038/srep41478 PMC5288656

[192] Montiel-Gonzalez MF, Vallecillo-Viejo I, Yudowski GA, Rosenthal JJC. Correction of mutations within the cystic fibrosis transmembrane conductance regulator by site-directed RNA editing. Proc Natl Acad Sci. 2013;110:18285–90. 24108353 10.1073/pnas.1306243110 PMC3831439

[193] Sinnamon JR, Kim SY, Corson GM, Song Z, Nakai H, Adelman JP, Mandel G. Site-directed RNA repair of endogenous Mecp2 RNA in neurons. Proc Natl Acad Sci. 2017;114:E9395–402. 29078406 10.1073/pnas.1715320114 PMC5676935

[194] Abudayyeh OO, Gootenberg JS, Franklin B, Koob J, Kellner MJ, Ladha A, Joung J, Kirchgatterer P, Cox DBT, Zhang F. A cytosine deaminase for programmable single-base RNA editing. Science. 2019;365:382–6. 31296651 10.1126/science.aax7063 PMC6956565

[195] Keeling KM, Xue X, Gunn G, Bedwell DM. Therapeutics based on stop codon readthrough. Annu Rev Genomics Hum Genet. 2014;15:371–94. 24773318 10.1146/annurev-genom-091212-153527 PMC5304456

[196] Brendel C, Belakhov V, Werner H, Wegener E, Gärtner J, Nudelman I, Baasov T, Huppke P. Readthrough of nonsense mutations in Rett syndrome: evaluation of novel aminoglycosides and generation of a new mouse model. J Mol Med. 2011;89:389–98. 21120444 10.1007/s00109-010-0704-4 PMC3055984

[197] Vecsler M, Zeev BB, Nudelman I, Anikster Y, Simon AJ, Amariglio N, Rechavi G, Baasov T, Gak E. *Ex Vivo* treatment with a novel synthetic aminoglycoside NB54 in primary fibroblasts from Rett syndrome patients suppresses MECP2 nonsense mutations. PLoS One. 2011;6:e20733. 21695138 10.1371/journal.pone.0020733 PMC3113846

[198] Wilhelm JM, Pettitt SE, Jessop JJ. Aminoglycoside antibiotics and eukaryotic protein synthesis: structure-function relationships in the stimulation of misreading with a wheat embryo system. Biochemistry. 1978;17:1143–9. 656378 10.1021/bi00600a001

[199] Palmer E, Wilhelm JM, Sherman F. Phenotypic suppression of nonsense mutants in yeast by aminoglycoside antibiotics. Nature. 1979;277:148–50. 366439 10.1038/277148a0

[200] Nau R, Sörgel F, Eiffert H. Penetration of drugs through the blood-cerebrospinal fluid/blood-brain barrier for treatment of central nervous system infections. Clin Microbiol Rev. 2010;23:858–83. 20930076 10.1128/CMR.00007-10 PMC2952976

[201] Csankovszki G, Nagy A, Jaenisch R. Synergism of Xist Rna, DNA methylation, and histone hypoacetylation in maintaining X chromosome inactivation. J Cell Biol. 2001;153:773–84. 11352938 10.1083/jcb.153.4.773 PMC2192370

[202] Mira-Bontenbal H, Tan B, Gontan C, et al. Genetic and epigenetic determinants of reactivation of Mecp2 and the inactive X chromosome in neural stem cells. Stem Cell Rep. 2022;17:693–706. 10.1016/j.stemcr.2022.01.008 PMC9039756 35148843

[203] Minajigi A, Froberg J, Wei C, et al. Chromosomes. A comprehensive Xist interactome reveals cohesin repulsion and an RNA-directed chromosome conformation. Science. 2015;349:10.1126/science.aab2276 aab2276. 26089354 10.1126/science.aab2276 PMC4845908

[204] Schulz EG, Meisig J, Nakamura T, Okamoto I, Sieber A, Picard C, Borensztein M, Saitou M, Blüthgen N, Heard E. The two active X chromosomes in female ESCs block exit from the pluripotent state by modulating the ESC signaling network. Cell Stem Cell. 2014;14:203–16. 24506884 10.1016/j.stem.2013.11.022

[205] Sripathy S, Leko V, Adrianse RL, et al. Screen for reactivation of MeCP2 on the inactive X chromosome identifies the BMP/TGF-β superfamily as a regulator of XIST expression. Proc Natl Acad Sci. 2017;114:1619–24. 28143937 10.1073/pnas.1621356114 PMC5321041

[206] Bhatnagar S, Zhu X, Ou J, Lin L, Chamberlain L, Zhu LJ, Wajapeyee N, Green MR. Genetic and pharmacological reactivation of the mammalian inactive X chromosome. Proc Natl Acad Sci U S A. 2014;111:12591–8. 25136103 10.1073/pnas.1413620111 PMC4156765

[207] Lee HM, Kuijer MB, Ruiz Blanes N, et al. A small-molecule screen reveals novel modulators of MeCP2 and X-chromosome inactivation maintenance. J Neurodev Disord. 2020;12:29. 33172406 10.1186/s11689-020-09332-3 PMC7657357

[208] Przanowski P, Wasko U, Zheng Z, Yu J, Sherman R, Zhu LJ, McConnell MJ, Tushir-Singh J, Green MR, Bhatnagar S. Pharmacological reactivation of inactive X-linked Mecp2 in cerebral cortical neurons of living mice. Proc Natl Acad Sci. 2018;115:7991–6. 30012595 10.1073/pnas.1803792115 PMC6077728

[209] Lee JT. Homozygous Tsix mutant mice reveal a sex-ratio distortion and revert to random X-inactivation. Nat Genet. 2002;32:195–200. 12145659 10.1038/ng939

[210] Huang H-S, Allen JA, Mabb AM, et al. Topoisomerase inhibitors unsilence the dormant allele of Ube3a in neurons. Nature. 2012;481:185–9. 10.1038/nature10726 PMC3257422 22190039

[211] Mitrea DM, Mittasch M, Gomes BF, Klein IA, Murcko MA. Modulating biomolecular condensates: a novel approach to drug discovery. Nat Rev Drug Discov. 2022;21:841–62. 35974095 10.1038/s41573-022-00505-4 PMC9380678

[212] Martin EW, Iserman C, Olety B, Mitrea DM, Klein IA. Biomolecular condensates as novel antiviral targets. J Mol Biol. 2024;436:168380. 38061626 10.1016/j.jmb.2023.168380

[213] Patel A, Mitrea D, Namasivayam V, Murcko MA, Wagner M, Klein IA. Principles and functions of condensate modifying drugs. Front Mol Biosci. 2022;9:1007744. 36483537 10.3389/fmolb.2022.1007744 PMC9725174

[214] Wyman J, Gill SJ. Ligand-linked phase changes in a biological system: applications to sickle cell hemoglobin. Proc Natl Acad Sci U S A. 1980;77:5239–42. 6933555 10.1073/pnas.77.9.5239 PMC350033

[215] Ruff KM, Dar F, Pappu RV. Polyphasic linkage and the impact of ligand binding on the regulation of biomolecular condensates. Biophys Rev (Melville). 2021;2:021302. 34179888 10.1063/5.0050059 PMC8211317

[216] Ruff KM, Dar F, Pappu RV. Ligand effects on phase separation of multivalent macromolecules. Proc Natl Acad Sci U S A. 2021;118:e2017184118. 33653957 10.1073/pnas.2017184118 PMC7958451

[217] Kilgore HR, Young RA. Learning the chemical grammar of biomolecular condensates. Nat Chem Biol. 2022;18:1298–306. 35761089 10.1038/s41589-022-01046-y PMC9691472

[218] Portz B, Lee BL, Shorter J. FUS and TDP-43 phases in health and disease. Trends Biochem Sci. 2021;46:550–63. 33446423 10.1016/j.tibs.2020.12.005 PMC8195841

[219] Taylor JP, Brown RH, Cleveland DW. Decoding ALS: from genes to mechanism. Nature. 2016;539:197–206. 27830784 10.1038/nature20413 PMC5585017

[220] Johnson BS, Snead D, Lee JJ, McCaffery JM, Shorter J, Gitler AD. TDP-43 is intrinsically aggregation-prone, and amyotrophic lateral sclerosis-linked mutations accelerate aggregation and increase toxicity. J Biol Chem. 2009;284:20329–39. 19465477 10.1074/jbc.M109.010264 PMC2740458

[221] Zacco E, Kantelberg O, Milanetti E, et al. Probing TDP-43 condensation using an in silico designed aptamer. Nat Commun. 2022;13:3306. 35739092 10.1038/s41467-022-30944-x PMC9226187

[222] Mann JR, Gleixner AM, Mauna JC, et al. RNA binding antagonizes neurotoxic phase transitions of TDP-43. Neuron. 2019;102:321-338.e8. 30826182 10.1016/j.neuron.2019.01.048 PMC6472983

[223] Babinchak WM, Dumm BK, Venus S, Boyko S, Putnam AA, Jankowsky E, Surewicz WK. Small molecules as potent biphasic modulators of protein liquid-liquid phase separation. Nat Commun. 2020;11:5574. 33149109 10.1038/s41467-020-19211-z PMC7643064

[224] Whyte WA, Orlando DA, Hnisz D, Abraham BJ, Lin CY, Kagey MH, Rahl PB, Lee TI, Young RA. Master transcription factors and mediator establish super-enhancers at key cell identity genes. Cell. 2013;153:307–19. 23582322 10.1016/j.cell.2013.03.035 PMC3653129

[225] Hnisz D, Abraham BJ, Lee TI, Lau A, Saint-André V, Sigova AA, Hoke HA, Young RA. Super-enhancers in the control of cell identity and disease. Cell. 2013;155:934–47. 24119843 10.1016/j.cell.2013.09.053 PMC3841062

[226] Klein IA, Boija A, Afeyan LK, et al. Partitioning of cancer therapeutics in nuclear condensates. Science. 2020;368:1386–92. 32554597 10.1126/science.aaz4427 PMC7735713

[227] Kathman SG, Koo SJ, Lindsey GL, et al. Remodeling oncogenic transcriptomes by small molecules targeting NONO. Nat Chem Biol. 2023;19:825–36. 36864190 10.1038/s41589-023-01270-0 PMC10337234

[228] Minkovsky A, Sahakyan A, Bonora G, Damoiseaux R, Dimitrova E, Rubbi L, Pellegrini M, Radu CG, Plath K. A high-throughput screen of inactive X chromosome reactivation identifies the enhancement of DNA demethylation by 5-aza-2’-dC upon inhibition of ribonucleotide reductase. Epigenetics Chromatin. 2015;8:42. 26468331 10.1186/s13072-015-0034-4 PMC4604769

[229] Collombet S, Rall I, Dugast-Darzacq C, Heckert A, Halavatyi A, Le Saux A, Dailey G, Darzacq X, Heard E. RNA polymerase II depletion from the inactive X chromosome territory is not mediated by physical compartmentalization. Nat Struct Mol Biol. 2023;30:1216–23. 37291424 10.1038/s41594-023-01008-5 PMC10442225

[230] Ditlev JA, Case LB, Rosen MK. Who’s in and who’s out—compositional control of biomolecular condensates. J Mol Biol. 2018;430:4666–84. 30099028 10.1016/j.jmb.2018.08.003 PMC6204295

[231] Wadsworth GM, Srinivasan S, Lai LB, Datta M, Gopalan V, Banerjee PR. RNA-driven phase transitions in biomolecular condensates. Mol Cell. 2024;84:3692–705. 39366355 10.1016/j.molcel.2024.09.005 PMC11604179

[232] Van Treeck B, Parker R. Emerging roles for intermolecular RNA-RNA interactions in RNP assemblies. Cell. 2018;174:791–802. 30096311 10.1016/j.cell.2018.07.023 PMC6200146

[233] Duszczyk MM, Sattler M. 1H, 13C, 15N and 31P chemical shift assignments of a human Xist RNA A-repeat tetraloop hairpin essential for X-chromosome inactivation. Biomol NMR Assign. 2012;6:75–7. 21779925 10.1007/s12104-011-9328-z

[234] Duszczyk MM, Wutz A, Rybin V, Sattler M. The Xist RNA A-repeat comprises a novel AUCG tetraloop fold and a platform for multimerization. RNA. 2011;17:1973–82. 21947263 10.1261/rna.2747411 PMC3198591

[235] Lu Z, Zhang QC, Lee B, et al. RNA duplex map in living cells reveals higher-order transcriptome structure. Cell. 2016;165:1267–79. 27180905 10.1016/j.cell.2016.04.028 PMC5029792

[236] Aguilar R, Spencer KB, Kesner B, et al. Targeting Xist with compounds that disrupt RNA structure and X inactivation. Nature. 2022;604:160–6. 35355011 10.1038/s41586-022-04537-z PMC11549687

[237] Warner KD, Hajdin CE, Weeks KM. Principles for targeting RNA with drug-like small molecules. Nat Rev Drug Discov. 2018;17:547–58. 29977051 10.1038/nrd.2018.93 PMC6420209

[238] Nickbarg EB, Spencer KB, Mortison JD, Lee JT. Targeting RNA with small molecules: lessons learned from Xist RNA. RNA. 2023;29:463–72. 36725318 10.1261/rna.079523.122 PMC10019374

[239] Palacino J, Swalley SE, Song C, et al. SMN2 splice modulators enhance U1-pre-mRNA association and rescue SMA mice. Nat Chem Biol. 2015;11:511–7. 26030728 10.1038/nchembio.1837

[240] Sivaramakrishnan M, McCarthy KD, Campagne S, et al. Binding to SMN2 pre-mRNA-protein complex elicits specificity for small molecule splicing modifiers. Nat Commun. 2017;8:1476. 29133793 10.1038/s41467-017-01559-4 PMC5684323

[241] Mukherjee S, Murata A, Ishida R, Sugai A, Dohno C, Hamada M, Krishna S, Nakatani K. HT-SELEX-based identification of binding pre-miRNA hairpin-motif for small molecules. Mol Ther Nucleic Acids. 2022;27:165–74. 34976435 10.1016/j.omtn.2021.11.021 PMC8685993

[242] Disney MD, Winkelsas AM, Velagapudi SP, Southern M, Fallahi M, Childs-Disney JL. Inforna 2.0: a platform for the sequence-based design of small molecules targeting structured RNAs. ACS Chem Biol. 2016;11:1720–8. 27097021 10.1021/acschembio.6b00001 PMC4912454

[243] Kovachka S, Panosetti M, Grimaldi B, Azoulay S, Di Giorgio A, Duca M. Small molecule approaches to targeting RNA. Nat Rev Chem. 2024;8:120–35. 38278932 10.1038/s41570-023-00569-9

[244] Padroni G, Patwardhan NN, Schapira M, Hargrove AE. Systematic analysis of the interactions driving small molecule-RNA recognition. RSC Med Chem. 2020;11:802–13. 33479676 10.1039/d0md00167h PMC7549050

[245] Vo DD, Tran TPA, Staedel C, Benhida R, Darfeuille F, Di Giorgio A, Duca M. Oncogenic MicroRNAs biogenesis as a drug target: structure-activity relationship studies on new aminoglycoside conjugates. Chemistry. 2016;22:5350–62. 26928593 10.1002/chem.201505094

[246] Aradi K, Di Giorgio A, Duca M. Aminoglycoside conjugation for RNA targeting: antimicrobials and beyond. Chemistry. 2020;26:12273–309. 32539167 10.1002/chem.202002258

[247] Dibrov SM, Ding K, Brunn ND, Parker MA, Bergdahl BM, Wyles DL, Hermann T. Structure of a hepatitis C virus RNA domain in complex with a translation inhibitor reveals a binding mode reminiscent of riboswitches. Proc Natl Acad Sci. 2012;109:5223–8. 22431596 10.1073/pnas.1118699109 PMC3325719

[248] Liang XH, Sun H, Shen W, et al. Antisense oligonucleotides targeting translation inhibitory elements in 5’ UTRs can selectively increase protein levels. Nucleic Acids Res. 2017;45:9528–46. 28934489 10.1093/nar/gkx632 PMC5766168

[249] Egli M, Manoharan M. Chemistry, structure and function of approved oligonucleotide therapeutics. Nucleic Acids Res. 2023;51:2529–73. 36881759 10.1093/nar/gkad067 PMC10085713

[250] Rinaldi C, Wood MJA. Antisense oligonucleotides: the next frontier for treatment of neurological disorders. Nat Rev Neurol. 2018;14:9–21. 29192260 10.1038/nrneurol.2017.148

[251] Roberts TC, Langer R, Wood MJA. Advances in oligonucleotide drug delivery. Nat Rev Drug Discov. 2020;19:673–94. 32782413 10.1038/s41573-020-0075-7 PMC7419031

[252] Liu X, Haniff HS, Childs-Disney JL, Shuster A, Aikawa H, Adibekian A, Disney MD. Targeted degradation of the oncogenic MicroRNA 17–92 cluster by structure-targeting ligands. J Am Chem Soc. 2020;142:6970–82. 32233464 10.1021/jacs.9b13159 PMC7357852

[253] Ries RJ, Zaccara S, Klein P, Olarerin-George A, Namkoong S, Pickering BF, Patil DP, Kwak H, Lee JH, Jaffrey SR. m6A enhances the phase separation potential of mRNA. Nature. 2019;571:424–8. 31292544 10.1038/s41586-019-1374-1 PMC6662915

[254] Wei G, Coker H, Rodermund L, Almeida M, Roach HL, Nesterova TB, Brockdorff N. N6-methyladenosine and the NEXT complex direct Xist RNA turnover and X inactivation dynamics. bioRxiv. 2025. 10.1101/2025.01.09.632176. 10.1038/s41594-025-01663-w PMC12618237 40926104

[255] Wang X, Liu C, Zhang S, et al. N6-methyladenosine modification of MALAT1 promotes metastasis via reshaping nuclear speckles. Dev Cell. 2021;56:702-715.e8. 33609462 10.1016/j.devcel.2021.01.015

[256] Fiorentino F, Menna M, Rotili D, Valente S, Mai A. METTL3 from target validation to the first small-molecule inhibitors: a medicinal chemistry journey. J Med Chem. 2023;66:1654–77. 36692498 10.1021/acs.jmedchem.2c01601 PMC9923689

[257] Bhattarai U, Hsieh WC, Yan H, Guo ZF, Shaikh AY, Soltani A, Song Y, Ly DH, Liang FS. Bifunctional small molecule-oligonucleotide hybrid as microRNA inhibitor. Bioorg Med Chem. 2020;28:115394. 32139203 10.1016/j.bmc.2020.115394 PMC9031885

[258] Nesterova TB, Wei G, Coker H, et al. Systematic allelic analysis defines the interplay of key pathways in X chromosome inactivation. Nat Commun. 2019;10:3129. 31311937 10.1038/s41467-019-11171-3 PMC6635394

[259] Lin Y, Protter DSW, Rosen MK, Parker R. Formation and maturation of phase-separated liquid droplets by RNA-binding proteins. Mol Cell. 2015;60:208–19. 26412307 10.1016/j.molcel.2015.08.018 PMC4609299

[260] Carey JL, Guo L. Liquid-liquid phase separation of TDP-43 and FUS in physiology and pathology of neurodegenerative diseases. Front Mol Biosci. 2022;9:826719. 35187086 10.3389/fmolb.2022.826719 PMC8847598

[261] Wiedner HJ, Giudice J. It’s not just a phase: function and characteristics of RNA-binding proteins in phase separation. Nat Struct Mol Biol. 2021;28:465–73. 34099940 10.1038/s41594-021-00601-w PMC8787349

[262] Petronilho EC, Pedrote MM, Marques MA, et al. Phase separation of p53 precedes aggregation and is affected by oncogenic mutations and ligands. Chem Sci. 2021;12:7334–49. 34163823 10.1039/d1sc01739j PMC8171334

[263] Krainer G, Welsh TJ, Joseph JA, et al. Reentrant liquid condensate phase of proteins is stabilized by hydrophobic and non-ionic interactions. Nat Commun. 2021;12:1085. 33597515 10.1038/s41467-021-21181-9 PMC7889641

[264] Heller GT, Shukla VK, Figueiredo AM, Hansen DF. Picosecond dynamics of a small molecule in its bound state with an intrinsically disordered protein. J Am Chem Soc. 2024;146:2319–24. 38251829 10.1021/jacs.3c11614 PMC10835725

[265] Fang MY, Markmiller S, Vu AQ, et al. Small-molecule modulation of TDP-43 recruitment to stress granules prevents persistent TDP-43 accumulation in ALS/FTD. Neuron. 2019;103:802-819.e11. 31272829 10.1016/j.neuron.2019.05.048 PMC6728177

[266] Yoshizawa T, Ali R, Jiou J, et al. Nuclear import receptor inhibits phase separation of FUS through binding to multiple sites. Cell. 2018;173:693-705.e22. 29677513 10.1016/j.cell.2018.03.003 PMC6234985

[267] Mukherjee H, Chan KP, Andresen V, Hanley ML, Gjertsen BT, Myers AG. Interactions of the natural product (+)-avrainvillamide with nucleophosmin and exportin-1 mediate the cellular localization of nucleophosmin and its AML-associated mutants. ACS Chem Biol. 2015;10:855–63. 25531824 10.1021/cb500872g PMC4652655

[268] Jégu T, Blum R, Cochrane JC, et al. Xist RNA antagonizes the SWI/SNF chromatin remodeler BRG1 on the inactive X chromosome. Nat Struct Mol Biol. 2019;26:96–109. 30664740 10.1038/s41594-018-0176-8 PMC6421574

[269] Lu S, Ye Q, Singh D, Cao Y, Diedrich JK, Yates JR, Villa E, Cleveland DW, Corbett KD. The SARS-CoV-2 nucleocapsid phosphoprotein forms mutually exclusive condensates with RNA and the membrane-associated M protein. Nat Commun. 2021;12:502. 33479198 10.1038/s41467-020-20768-y PMC7820290

[270] Wang S, Dai T, Qin Z, et al. Targeting liquid-liquid phase separation of SARS-CoV-2 nucleocapsid protein promotes innate antiviral immunity by elevating MAVS activity. Nat Cell Biol. 2021;23:718–32. 34239064 10.1038/s41556-021-00710-0

[271] Burslem GM, Crews CM. Proteolysis-targeting chimeras as therapeutics and tools for biological discovery. Cell. 2020;181:102–14. 31955850 10.1016/j.cell.2019.11.031 PMC7319047

[272] Békés M, Langley DR, Crews CM. PROTAC targeted protein degraders: the past is prologue. Nat Rev Drug Discov. 2022;21:181–200. 35042991 10.1038/s41573-021-00371-6 PMC8765495

[273] Hyun S, Shin D. Chemical-mediated targeted protein degradation in neurodegenerative diseases. Life (Basel). 2021;11:607. 34202541 10.3390/life11070607 PMC8305580

[274] Gao N, Huang YP, Chu TT, Li QQ, Zhou B, Chen YX, Zhao YF, Li YM. TDP-43 specific reduction induced by Di-hydrophobic tags conjugated peptides. Bioorg Chem. 2019;84:254–9. 30508770 10.1016/j.bioorg.2018.11.042

[275] Qu J, Ren X, Xue F, He Y, Zhang R, Zheng Y, Huang H, Wang W, Zhang J. Specific knockdown of α-Synuclein by peptide-directed proteasome degradation rescued its associated neurotoxicity. Cell Chem Biol. 2020;27:751-762.e4. 32359427 10.1016/j.chembiol.2020.03.010

[276] Silva MC, Ferguson FM, Cai Q, et al. Targeted degradation of aberrant tau in frontotemporal dementia patient-derived neuronal cell models. Elife. 2019;8:e45457. 30907729 10.7554/eLife.45457 PMC6450673

[277] Zhang X, Crowley VM, Wucherpfennig TG, Dix MM, Cravatt BF. Electrophilic PROTACs that degrade nuclear proteins by engaging DCAF16. Nat Chem Biol. 2019;15:737–46. 31209349 10.1038/s41589-019-0279-5 PMC6592777

[278] Yu X, Hu W, Dong H, Zhao T, Wang X, Chen L, Xue S, Li JP, Luo SZ. Phase separation enhanced PROTAC for highly efficient protein degradation. Biomacromol. 2024;25:4374–83. 10.1021/acs.biomac.4c00424 38825770

[279] Sanders DW, Kedersha N, Lee DSW, et al. Competing protein-RNA interaction networks control multiphase intracellular organization. Cell. 2020;181:306-324.e28. 32302570 10.1016/j.cell.2020.03.050 PMC7816278

[280] Gerry CJ, Schreiber SL. Unifying principles of bifunctional, proximity-inducing small molecules. Nat Chem Biol. 2020;16:369–78. 32198490 10.1038/s41589-020-0469-1 PMC7312755

[281] Dao TP, Kolaitis RM, Kim HJ, O’Donovan K, Martyniak B, Colicino E, Hehnly H, Taylor JP, Castañeda CA. Ubiquitin modulates liquid-liquid phase separation of UBQLN2 via disruption of multivalent interactions. Mol Cell. 2018;69:965-978.e6. 29526694 10.1016/j.molcel.2018.02.004 PMC6181577

[282] Monahan Z, Ryan VH, Janke AM, et al. Phosphorylation of the FUS low-complexity domain disrupts phase separation, aggregation, and toxicity. EMBO J. 2017;36:2951–67. 28790177 10.15252/embj.201696394 PMC5641905

[283] Snead WT, Gladfelter AS. The control centers of biomolecular phase separation: how membrane surfaces, PTMs, and active processes regulate condensation. Mol Cell. 2019;76:295–305. 31604601 10.1016/j.molcel.2019.09.016 PMC7173186

[284] Guo YE, Manteiga JC, Henninger JE, et al. Pol II phosphorylation regulates a switch between transcriptional and splicing condensates. Nature. 2019;572:543–8. 31391587 10.1038/s41586-019-1464-0 PMC6706314

[285] Conti BA, Oppikofer M. Biomolecular condensates: new opportunities for drug discovery and RNA therapeutics. Trends Pharmacol Sci. 2022;43:820–37. 36028355 10.1016/j.tips.2022.07.001

[286] Uechi H, Sridharan S, Nijssen J, et al. Small molecule modulation of a redox-sensitive stress granule protein dissolves stress granules with beneficial outcomes for familial amyotrophic lateral sclerosis models. bioRxiv. 2024. 10.1101/2024.01.01.721001.

[287] Hernández-Candia CN, Pearce S, Tucker CL. A modular tool to query and inducibly disrupt biomolecular condensates. Nat Commun. 2021;12:1809. 33753744 10.1038/s41467-021-22096-1 PMC7985322

[288] Bhat P, Honson D, Guttman M. Nuclear compartmentalization as a mechanism of quantitative control of gene expression. Nat Rev Mol Cell Biol. 2021;22:653–70. 34341548 10.1038/s41580-021-00387-1 PMC12145136

[289] Marahrens Y, Panning B, Dausman J, Strauss W, Jaenisch R. Xist-deficient mice are defective in dosage compensation but not spermatogenesis. Genes Dev. 1997;11:156–66. 9009199 10.1101/gad.11.2.156

[290] Grimm NB, Lee JT. Selective Xi reactivation and alternative methods to restore MECP2 function in Rett syndrome. Trends Genet. 2022;38:920–43. 35248405 10.1016/j.tig.2022.01.007 PMC9915138

[291] Yang L, Kirby JE, Sunwoo H, Lee JT. Female mice lacking Xist RNA show partial dosage compensation and survive to term. Genes Dev. 2016;30:1747–60. 27542829 10.1101/gad.281162.116 PMC5002979

[292] Lee JT. Gracefully ageing at 50, X-chromosome inactivation becomes a paradigm for RNA and chromatin control. Nat Rev Mol Cell Biol. 2011;12:815–26. 22108600 10.1038/nrm3231

[293] Risso-Ballester J, Galloux M, Cao J, et al. A condensate-hardening drug blocks RSV replication *in vivo*. Nature. 2021;595:596–9. 34234347 10.1038/s41586-021-03703-z

[294] Erwin GS, Grieshop MP, Ali A, et al. Synthetic transcription elongation factors license transcription across repressive chromatin. Science. 2017;358:1617–22. 29192133 10.1126/science.aan6414 PMC6037176

