# Supplementary material for: Xist condensates: perspectives for therapeutic intervention
Source: Genome Biol. 2025 Jul 21;26:215. doi: 10.1186/s13059-025-03666-8 (PMC12278583; doi:10.1186/s13059-025-03666-8)
Supplement: Supplementary file 1 — Additional file 1: Table S1. X-Linked Dominant Diseases as Potential Targets for Xi Reactivation. This table lists ten X-linked dominant disorders that may represent potential therapeutic targets for reactivation of the inactive X chromosome (Xi), providing disease names and relevant context. [file 13059_2025_3666_MOESM1_ESM.docx]

| **Disease** | ***Locus*** ^1^ | **Clinical Features**^1^ | **Mutation** | **Population** | **Role** | **XCI**^1,2^ | **Restorative approaches** |
| --- | --- | --- | --- | --- | --- | --- | --- |
| **Rett Syndrome** | *MECP2* | Loss of motor skills, intellectual disability, seizures and hand-wringing movements | - Missense  - Nonsense  - Frameshift  - Splice-site  - Start codon | ~1/10,000 to 15,000 | TF | Random | Functional restoration ^3–6^, DNA/RNA editing ^7–9^,  Non-sense readthrough therapies ^10–13^, Xi reactivation ^14,15^ |
| **Oral–facial–digital type I** | *OFD1* | Cleft palate, wide-set eyes, polydactyly and developmental delays, hearing loss and brain, heart kidneys structural issues | - Deletions  - Insertions  - [Missense](https://www.ncbi.nlm.nih.gov/books/n/gene/glossary/def-item/missense/)  - Nonsense  - Splice-site | 1/250,000 to 1/50,000 | Centrosomal protein | Non-random  in 30% | N.A. |
| **Microphthalmia with linear skin-defects** | *MLS* | Small eyes, linear skin defects, possible intellectual disability, potential hearing loss, heart defects, and growth delays | - Deletions  - Translocations | Extremely rare | Cell metabolism | Skewed | N.A. |
| **Goltz syndrome** | *FDH* | Skin manifestations, eyes, teeth, skeleton, urinary tract, gastrointestinal, cardiovascular, and central nervous system abnormalities | - Nonsense  - Missense  - Frameshift  - Deletions | ~300 | Regulator of Wnt signaling | Skewed | N.A. |
| **Incontinentia Pigmenti** | *IP* | Blistering, hyperpigmentation, and eyes, teeth, skeleton, heart, and central nervous system abnormalities | - [Missense](https://www.ncbi.nlm.nih.gov/books/n/gene/glossary/def-item/missense/)  - Frameshift  - Nonsense  - Splice-site | 1/143,000 | IKBKG inhibitor of nuclear factor kB kinase b subunit | Skewed | N.A. |
| **Aicardi Syndrome** | *AIC* | Agenesis of corpus callosum, seizures, intellectual disability and retinal abnormalities | N.A. | ~200 | N.A. | Likely  random | N.A. |
| **Terminal osseous dysplasia and pigmentary defects** | *ODPD* | Skeletal dysplasia, skin pigmentary defects, recurrent digital fibromatosis, delayed ossification, joint contractures, and dysmorphic facial features | N.A. | ~1/1,000000 | N.A. | Skewed | N.A. |
| **Oculo-facio-cardio-dental Syndrome** | *OFCD* | Facial abnormalities, cataracts, microphthalmia, dental abnormalities, and cardiac septal defects | - Missense  - Frameshift  - Deletion | Extremely rare | Epigenetic regulator | Skewed | N.A. |
| **Congenital hemidysplasia** with **ichthyosiform erythroderma** and **limb *defectsa*** | *CHILD* | Manifestations on the skin and underlying musculoskeletal structures | - Missense  - Nonsense | ~1/100,000 | NAD(P)-dependent steroid dehydrogenase-like protein | Random | N.A. |
| **Chondrodysplasia punctata 2** | *CDPX2* | Skin defects, skeletal abnormalities, epiphyseal stippling, and craniofacial malformations | - Deletions  - Insertions  - Missense  - Nonsense | ~1/400,000 | Emopamil binding protein involved in cholesterol biosynthesis | Random | N.A. |
| **CDKL5 disorder** | *CDKL5* | Early onset of epileptic encephalopathy, infantile spasms, seizures autistic phenotype,  inability to walk or talk  and visual impairment | - Missense  - Nonsense  - Frameshift  - Splice-site  - Deletions | 1/45,000 | Protein kinase  (in brain) | Random | Non-sense readthrough therapies ^16^,  Gene restoration ^17,18^,  Xi reactivation ^19^07/02/2025 17:19:00 |

**Additional file 1: Table S1. X-Linked Dominant Diseases as Potential Targets for Xi Reactivation**

This table presents ten different X-linked dominant disorders. Information on clinical features, underlying mutations, and affected populations were obtained from publicly available repositories (*e.g*., https://www.orpha.net/, <https://www.nih.gov/health-information>. TF, transcription factor; XCI, X Chromosome Inactivation in patients. ^c^ Reported restorative approaches to date; XCR, X chromosome reactivation. Note that mutations in CDKL5 typically result in a lethal disorder in males.

**References relative to Additional file 1: Table S1 are listed below and cited in the main text of the review**

1. Franco, B., and Ballabio, A. (2006). X-inactivation and human disease: X-linked dominant male-lethal disorders. Curr Opin Genet Dev *16*, 254–259. https://doi.org/10.1016/j.gde.2006.04.012.

2. Przanowski, P., Wasko, U., Zheng, Z., Yu, J., Sherman, R., Zhu, L.J., McConnell, M.J., Tushir-Singh, J., Green, M.R., and Bhatnagar, S. (2018). Pharmacological reactivation of inactive X-linked Mecp2 in cerebral cortical neurons of living mice. Proceedings of the National Academy of Sciences *115*, 7991–7996. https://doi.org/10.1073/pnas.1803792115.

3. Dimitrov, D.S. (2012). Therapeutic proteins. Methods Mol Biol *899*, 1–26. https://doi.org/10.1007/978-1-61779-921-1_1.

4. Powers, S., Miranda, C., Dennys-Rivers, C., Huffenberger, A., Braun, L., Rinaldi, F., Wein, N., Meyer, K.C., Solano, S., Nguyen, K., et al. (2019). Rett syndrome gene therapy improves survival and ameliorates behavioral phenotypes in MeCP2 null (S51.002). Neurology *92*, S51.002. https://doi.org/10.1212/WNL.92.15_supplement.S51.002.

5. Sinnett, S.E., Boyle, E., Lyons, C., and Gray, S.J. (2021). Engineered microRNA-based regulatory element permits safe high-dose miniMECP2 gene therapy in Rett mice. Brain *144*, 3005–3019. https://doi.org/10.1093/brain/awab182.

6. Gadalla, K.K.E., Bailey, M.E.S., Spike, R.C., Ross, P.D., Woodard, K.T., Kalburgi, S.N., Bachaboina, L., Deng, J.V., West, A.E., Samulski, R.J., et al. (2013). Improved survival and reduced phenotypic severity following AAV9/MECP2 gene transfer to neonatal and juvenile male Mecp2 knockout mice. Mol Ther *21*, 18–30. https://doi.org/10.1038/mt.2012.200.

7. Croci, S., Carriero, M.L., Capitani, K., Daga, S., Donati, F., Frullanti, E., Lamacchia, V., Tita, R., Giliberti, A., Valentino, F., et al. (2020). High rate of HDR in gene editing of p.(Thr158Met) MECP2 mutational hotspot. Eur J Hum Genet *28*, 1231–1242. https://doi.org/10.1038/s41431-020-0624-x.

8. Sinnamon, J.R., Kim, S.Y., Corson, G.M., Song, Z., Nakai, H., Adelman, J.P., and Mandel, G. (2017). Site-directed RNA repair of endogenous Mecp2 RNA in neurons. Proceedings of the National Academy of Sciences *114*, E9395–E9402. https://doi.org/10.1073/pnas.1715320114.

9. Sinnamon, J.R., Kim, S.Y., Fisk, J.R., Song, Z., Nakai, H., Jeng, S., McWeeney, S.K., and Mandel, G. (2020). In Vivo Repair of a Protein Underlying a Neurological Disorder by Programmable RNA Editing. Cell Rep *32*, 107878. https://doi.org/10.1016/j.celrep.2020.107878.

10. Brendel, C., Belakhov, V., Werner, H., Wegener, E., Gärtner, J., Nudelman, I., Baasov, T., and Huppke, P. (2011). Readthrough of nonsense mutations in Rett syndrome: evaluation of novel aminoglycosides and generation of a new mouse model. J Mol Med *89*, 389–398. https://doi.org/10.1007/s00109-010-0704-4.

11. Vecsler, M., Zeev, B.B., Nudelman, I., Anikster, Y., Simon, A.J., Amariglio, N., Rechavi, G., Baasov, T., and Gak, E. (2011). Ex Vivo Treatment with a Novel Synthetic Aminoglycoside NB54 in Primary Fibroblasts from Rett Syndrome Patients Suppresses MECP2 Nonsense Mutations. PLOS ONE *6*, e20733. https://doi.org/10.1371/journal.pone.0020733.

12. Pitcher, M.R., Herrera, J.A., Buffington, S.A., Kochukov, M.Y., Merritt, J.K., Fisher, A.R., Schanen, N.C., Costa-Mattioli, M., and Neul, J.L. (2015). Rett syndrome like phenotypes in the R255X Mecp2 mutant mouse are rescued by MECP2 transgene. Hum Mol Genet *24*, 2662–2672. https://doi.org/10.1093/hmg/ddv030.

13. Popescu, A.C., Sidorova, E., Zhang, G., and Eubanks, J.H. (2010). Aminoglycoside-mediated partial suppression of MECP2 nonsense mutations responsible for Rett syndrome in vitro. J Neurosci Res *88*, 2316–2324. https://doi.org/10.1002/jnr.22409.

14. Carrette, L.L.G., Wang, C.-Y., Wei, C., Press, W., Ma, W., Kelleher, R.J., and Lee, J.T. (2018). A mixed modality approach towards Xi reactivation for Rett syndrome and other X-linked disorders. Proc Natl Acad Sci U S A *115*, E668–E675. https://doi.org/10.1073/pnas.1715124115.

15. Mira-Bontenbal, H., Tan, B., Gontan, C., Goossens, S., Boers, R.G., Boers, J.B., Dupont, C., van Royen, M.E., IJcken, W.F.J., French, P., et al. (2022). Genetic and epigenetic determinants of reactivation of Mecp2 and the inactive X chromosome in neural stem cells. Stem Cell Reports *17*, 693–706. https://doi.org/10.1016/j.stemcr.2022.01.008.

16. Fazzari, M., Frasca, A., Bifari, F., and Landsberger, N. (2019). Aminoglycoside drugs induce efficient read-through of CDKL5 nonsense mutations, slightly restoring its kinase activity. RNA Biology *16*, 1414–1423. https://doi.org/10.1080/15476286.2019.1632633.

17. Voronin, G., Narasimhan, J., Gittens, J., Sheedy, J., Lipari, P., Peters, M., DeMarco, S., Cao, L., Varganov, Y., Kim, M.J., et al. (2024). Preclinical studies of gene replacement therapy for CDKL5 deficiency disorder. Mol Ther *32*, 3331–3345. https://doi.org/10.1016/j.ymthe.2024.07.012.

18. Medici, G., Tassinari, M., Galvani, G., Bastianini, S., Gennaccaro, L., Loi, M., Mottolese, N., Alvente, S., Berteotti, C., Sagona, G., et al. (2022). Expression of a Secretable, Cell-Penetrating CDKL5 Protein Enhances the Efficacy of Gene Therapy for CDKL5 Deficiency Disorder. Neurotherapeutics *19*, 1886–1904. https://doi.org/10.1007/s13311-022-01295-8.

19. Halmai, J.A.N.M., Deng, P., Gonzalez, C.E., Coggins, N.B., Cameron, D., Carter, J.L., Buchanan, F.K.B., Waldo, J.J., Lock, S.R., Anderson, J.D., et al. (2020). Artificial escape from XCI by DNA methylation editing of the CDKL5 gene. Nucleic Acids Research *48*, 2372–2387. https://doi.org/10.1093/nar/gkz1214.
